# Chemosensors based on N-heterocyclic dyes: advances in sensing highly toxic ions such as CN^−^ and Hg^2+^

**DOI:** 10.1039/d1ra06567j

**Published:** 2021-10-21

**Authors:** María-Camila Ríos, Néstor-Fabián Bravo, Christian-Camilo Sánchez, Jaime Portilla

**Affiliations:** Bioorganic Compounds Research Group, Department of Chemistry, Universidad de los Andes Carrera 1 No. 18A-10 Bogotá 111711 Colombia jportill@uniandes.edu.co

## Abstract

CN^−^ and Hg^2+^ ions are harmful to both the environment and human health, even at trace levels. Thus, alternative methods for their detection and quantification are highly desirable given that the traditional monitoring systems are expensive and require qualified personnel. Optical chemosensors (probes) have revolutionized the sensing of different species due to their high specificity and sensitivity, corresponding with their modular design. They have also been used in aqueous media and different pH ranges, facilitating their applications in various samples. The design of molecular probes is based on organic dyes, where the key species are N-heterocyclic compounds (NHCs) due to their proven photophysical properties, biocompatibility, and synthetic versatility, which favor diverse applications. Accordingly, this review aims to provide an overview of the reports from 2016 to 2021, in which fluorescent probes based on five- and six-membered N-heterocycles are used for the detection of CN^−^ and Hg^2+^ ions.

## Abstract 2

This review considers the most recent advances in sensing highly toxic ions such as CN^−^ and Hg^2+^. Specifically, probes bearing N-heterocyclic compounds for their proven versatility for applications in the development of chemosensors are analyzed.

## Graphical abstract

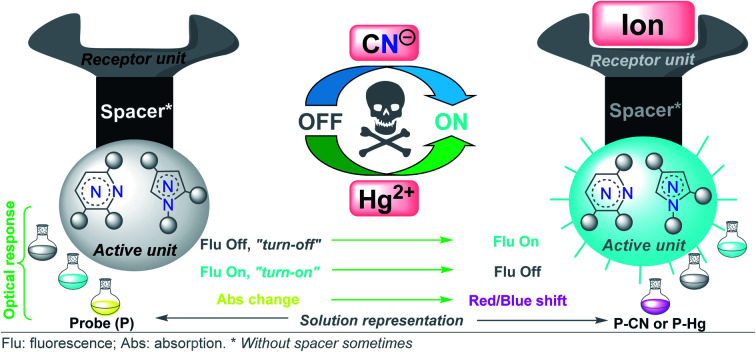

## 1. Introduction

**Fig. 1:**
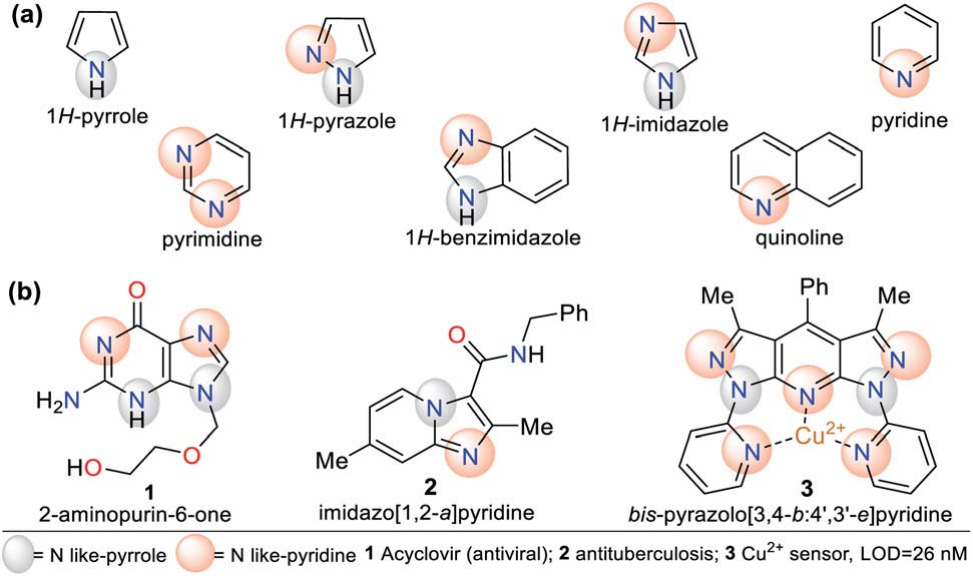
Structure of (a) five- and six-membered N-heterocycles and (b) some representative examples.

N-Heterocyclic compounds (NHCs) are molecules in which nitrogen atoms (N) replace one or more carbon atoms in their ring. These compounds have a nucleophilic or basic character due to the unbound electrons pair in the nitrogen atom-like-pyrrole (–N) or -pyridine (=N), respectively.^1,2^ In saturated NHCs, the heteroatom behaves similarly to that in alkylamines; however, in heteroaromatic rings, the N-like-pyrrole contributes to the π-conjugation and allows special electronic properties, which depend on the substituents and fused or rigid structural nature of the rings.^1–4^ Various NHCs are present in natural products such as nucleic acids, coenzymes, amino acids, and alkaloids.^1–6^ NHCs are classified based on the number of bonds and nitrogen atoms in their ring, from three members onwards. Five- and six-membered rings such as pyrroles, imidazoles, pyrazoles, pyridines, and pyrimidines are more common due to their high stability and applicability. These compounds can also be classified based on their saturation or fusion with other rings, *e.g.*, benzimidazole and quinoline are benzo-fused cores of five- and six-membered rings, respectively (Fig. 1a).^1–8^

N-Heterocyclic systems have many applications due to their high synthetic versatility and significant structural diversity obtained by varying their substituent or functional groups. These applications include relevant biological effects such as anticoagulant,^9^ anti-inflammatory,^10^ antimicrobial,^11,12^ and anticancer^13^ agents. Additionally, they can be applied in photophysical and materials sciences. N-Heterocycles have been applied for the design and synthesis of new luminescent compounds such as organic light-emitting diodes (OLEDs),^14^ technological devices,^15^ hybrid-phosphorescent materials,^16^ and more frequently, fluorescent probes for the detection of different species^17–20^ (Fig. 1b).

### 1.1. Photophysical features of N-heterocycles

The physicochemical properties of N-heteroaromatic systems are unique given that saturated derivatives behave analogous to open-chain compounds.^1,2^ Thus, NHCs tend to be conjugated molecules with unique and intrinsic photophysical properties, which can be employed for the development of sensors and organic materials. For instance, porphyrins (pyrrole tetramer) have high absorption in the visible spectrum red region and are potential dyes for sensitizers in photodynamic therapy. This feature promotes studies on the synthesis of porphyrins bearing aromatic substituents on their rings to extend their π-conjugation. The inclusion of chromophore groups in the porphyrinic ring enhances its photophysical properties and versatility, resulting in a shift in absorption wavelength. Attractive examples of π-extended conjugation porphyrins are systems in which aryl and acenaphthylene groups are adequately incorporated.^1,21^

**Fig. 2:**
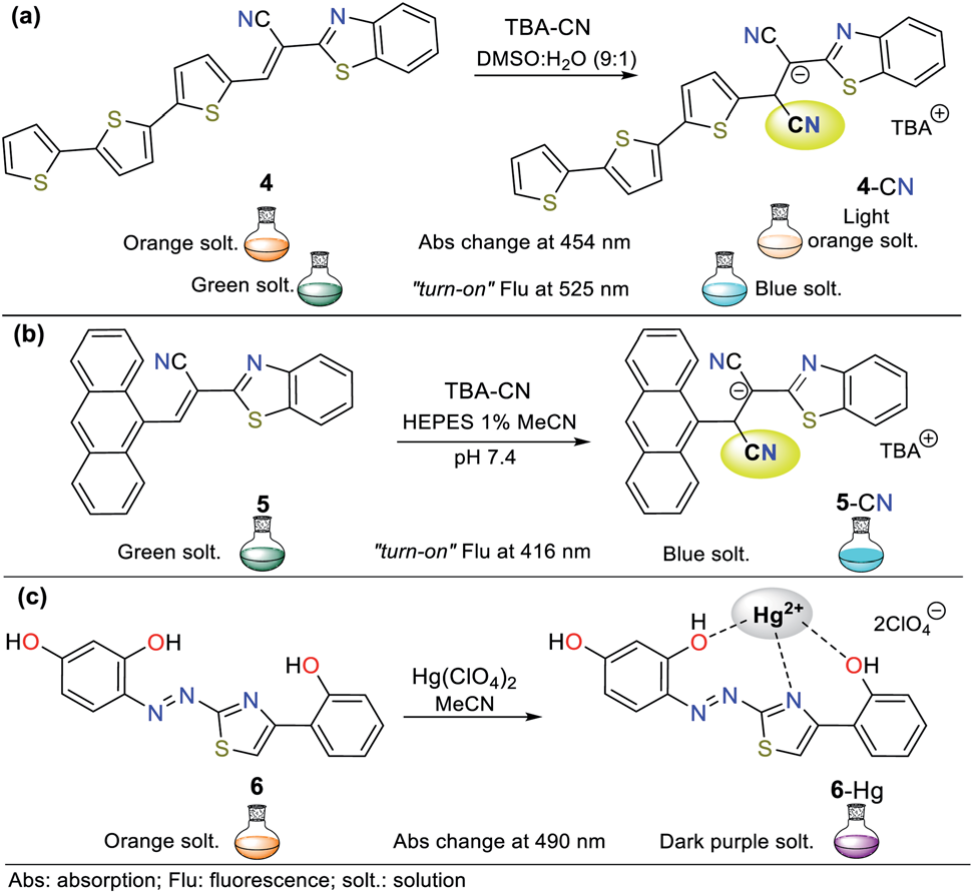
Probes based on thiazoles for CN^−^ sensing in (a) DMSO : H_2_O and (b) in HEPES solution (1% MeCN as a co-solvent), and (c) for Hg^2+^ recognition.

Regarding probes bearing an N-heterocyclic core that distinguish cyanide (CN^−^) and mercury(ii) (Hg^2+^) ions, obviously they include those possessing other heteroatoms besides nitrogen. Indeed, the thiazole ring has nitrogen and sulfur atoms, and its derivatives have been used for this purpose. The oligothiophene–benzothiazole 4 is a colorimetric and fluorimetric probe for sensing CN^−^ with a limit of detection (LOD) of 4.60 × 10^−7^ M in DMSO : H_2_O (9 : 1), which acts *via* a nucleophilic addition reaction on the electrophilic cyanovinylidene group of 4. Colorimetric studies showed that 4 has an absorption band at 349 nm attributed to the π–π* transitions and another at 485 nm due to the internal charge transfer (ICT) process (Fig. 2a).^22^

Upon the addition of CN^−^ to a solution of 4, the band at 485 nm decreases with the appearance of a new band at 353 nm and an isosbestic point at 411 nm accompanied with a color change from orange to colorless. Besides, emission studies showed a color change from pale green to fluorescent blue with the addition of CN^−^. The spectra for the titration showed a blue-shift in its fluorescence band from 560 nm to 525 nm (*λ*_exc_ = 353 nm). The color changes occur due to the interruption of both the π-conjugation and ICT process (Fig. 2a).^22^ Similarly, Vidya *et al.*^23^ developed chemodosimeter 5 for sensing CN^−^ (LOD = 5.52 × 10^−8^ M), working *via* “*turn-on*” fluorescence in a ratiometric manner (Fig. 2b). This probe also acts *via* the disruption of the ICT process, where in this case, it occurs between the anthracene and benzothiazole moieties.

Alternatively, Yeap and co-workers^24^ synthesized the resorcinol derivative 6 bearing the 2-thiazolyldiazenyl group at position 4, which works for the colorimetric detection of Hg^2+^ (LOD = 1.20 × 10^−7^ M in MeCN). This probe shows two absorption bands at 350 and 465 nm, which are assigned to the n–π* and π–π* transitions of the aryl-azo group and the thiazole ring, respectively (Fig. 2c). Upon the addition of Hg^2+^ to the solution of 6 in acetonitrile, a new band appears at 490 nm, changing from orange to purple. The redshifted band (465 nm) decreased with an increase in the cation concentration, while a new band appeared and increased, accompanied by an isosbestic point at 476 nm.

Although probes 4 to 6 work well for the detection of CN^−^ and Hg^2+^, their N-heterocyclic cores also possess a sulfur heteroatom. Thus, these probes use a signaling unit based on different heteroatoms (N and S); however, the probes discussed throughout this review have a signaling unit based mainly on rings bearing only one type of heteroatom, *i.e.*, one or two nitrogen atoms. Additionally, both five- and six-membered rings considered herein are not only found as chromophores or fluorophores but as fused systems or high π-conjugation molecules. This is due to the synthetic and photophysical versatility of NHCs, which allow their good functionalization and utility in ion detection, respectively. Ultimately, because of this, we managed to focus our discussion on the strategic rings investigated for the recognition of different species.

**Fig. 3:**
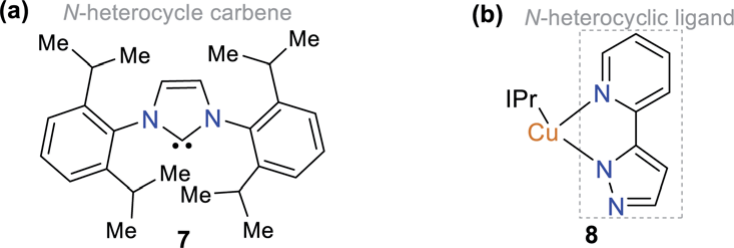
Structure of (a) ligand IPr 7 and (b) Cu(i) complex 8.

Some NHCs have been used as ligands of organometallic and coordination compounds. Their combination with metals can modify their charge transfer features and some photophysical properties in the resulting complex.^18,25^ For example, complexes with carbene derivatives such as 1,3-bis(2,6-diisopropylphenyl)imidazol-2-yl-idene (IPr, structure 7) and coordinating metals such as copper(i) (complex 8) show significant photophysical properties (Fig. 3).^25,26^ Regarding coordination complexes, some derivatives with *N*,*N*-donor ligands are notable optical materials.^27^ Thus, complexes based on N-heterocyclic compounds represent a good option for the development of luminescent materials and applications related to fluorescence properties such as molecular organic electronics and monitoring applications in both environmental and biological systems.^28–31^

**Fig. 4:**
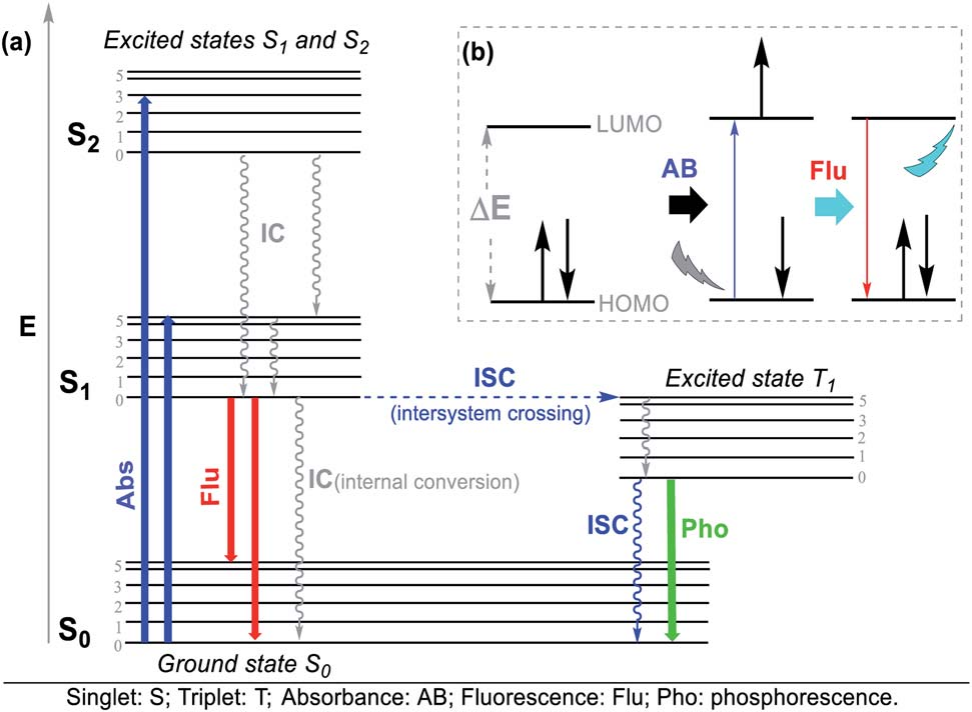
(a) Jablonski diagram and (b) photophysical process.

The Jablonski diagram (Fig. 4a) is suitable to interpret the molecular photophysical routes (Fig. 4b) given that it shows the energies of the electronic states and the interconversion processes. This diagram displays two photophysical pathways, *i.e.*, the radiative (indicated by straight lines) and non-radiative (denoted by wavy lines) pathways. The former occurs *via* the absorption or emission of light, whereas the latter occurs without these routes.^32^ Absorbance (Abs), fluorescence (Flu), and phosphorescence (Pho) are the typical photophysical processes occurring on NHCs. Abs (blue line) involves one-electron excitation from the highest occupied molecular orbital (HOMO) to the lowest unoccupied molecular orbital (LUMO) in a dye molecule, that is, a transition from the ground state to the excited state. In the Flu process (red line), photon emission is accompanied by a transition from the excited state to the ground state without a change in multiplicity. The Pho processes (green line) are the emission from photons accompanied by a transition from the excited state to the ground state, where a change in multiplicity usually occurs from the triplet excited state to singlet ground state (Fig. 4).^32^

**Fig. 5:**
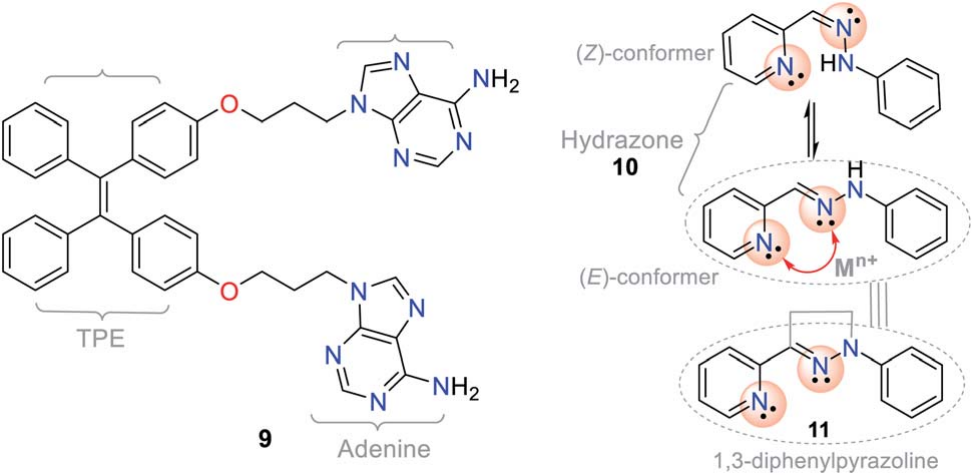
Structures of the hybrid system 9, hydrazone 10, and pyrazoline 11.

The different photophysical pathways allow NHCs to be employed as strategic molecules for sensing studies *via* mechanisms such as ICT, twisted intramolecular charge transfer (TICT), and photoinduced electron transfer (PET). The synthetic versatility provided by these compounds for the modification of their photophysical properties makes them a good option for sensing systems, and accordingly, for the detection of analytes. For instance, the bis-adenine–tetraphenylethylene (TPE) hybrid system (probe 9) showed fluorescence “*turn-on*” in the presence of Ag^+^*via* the aggregation-induced emission (AIE) phenomenon (Fig. 5),^17,18,20,33,34^ in which the adenine-mediated coordination of the silver ions induced the aggregation of TPE, resulting in a fluorescent system.^34^

Another example involving NHCs is the isomerization of the hydrazone (C=N–N) moiety, where the signaling route originates from the conformationally restricted molecules, as shown in the photophysical study. For instance, hydrazone 10 has a freely rotating C=N bond, while pyrazoline (cyclic hydrazone) 11 has restricted rotation. When hydrazones are isomerized, their fluorescence is low or non-existent. Nevertheless, several pyrazoline derivatives show high fluorescence intensities, as evidenced in PET “*turn-on*” processes (PET-ON) when the isomerization of some hydrazones is restricted in the presence of metal ions (Fig. 5, red arrow).^35^ In hydrazone-free derivatives, this conformational phenomenon occurs because the isomerization is the principal decay process of the excited states, making these molecules non-fluorescent.^33^

Exited state intramolecular proton transfer (ESIPT) is a notable process that also involves the above-mentioned phenomenon, in which the protons of the system involved in the excited state depart or join a molecule at different rates to that in the ground state, for instance, the fluorescent properties incorporate the ground and excited states of two different tautomers. This process usually involves the transfer of a proton from a hydroxyl (OH) or amino (NH_2_) group to a carbonyl oxygen (C=O) or imine nitrogen (C=N) that are no more than 2 Å apart. Thus, it can be expected that molecular probes for “*turn-on*” or “*turn-off*” fluorescence^33^ based on NHCs will be useful.

### 1.2. Chemosensors for the detection of CN^−^ and Hg^2+^

Chemical detection *via* colorimetry or fluorimetry is a promising qualitative and quantitative method for ion or molecule sensing in the environment and medicine. The advantages of this method include the use of simple equipment, short-term detection, high selectivity and sensitivity, and avoiding sample pretreatment.^17,18,20,24,36–39^ Numerous screening tests are performed with the standard atomic absorption (AA) methods and inductively coupled plasma emission spectroscopy (ICP-AES) to quantify different ions with high efficiency and at trace levels.^36–39^ The rapidly developed molecular sensor chemistry strongly relates to photochemistry given that the probe interaction with the analyte results in variations in their photophysical properties and spectral changes in the chemosensor–analyte complexes.^40^

**Fig. 6:**
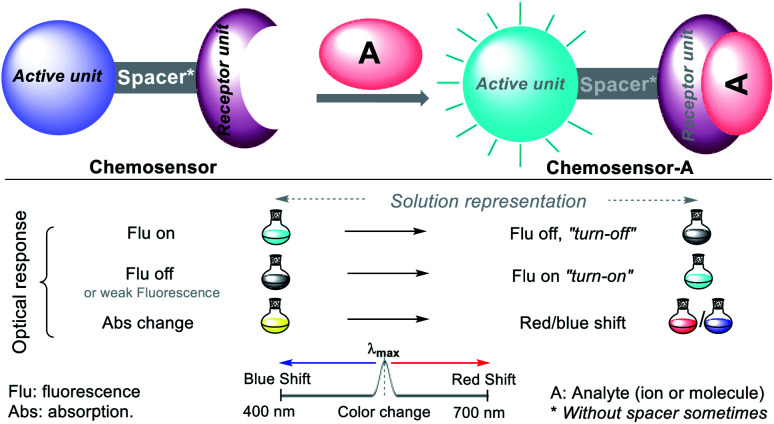
Conventional configuration and optical response of a chemosensor.

A chemosensor is a chemical species capable of transforming a molecular change into a measurable analytical signal (color or fluorescence). This variation generates electronic changes, and thus photophysical changes in the host molecule, promoting the signal in the chromophore or fluorophore unit of the chemosensor. Precisely, the probe consists of a receptor moiety, which is responsible for the selective binding of the analyte (ion or molecule). The properties of this photoactive unit change upon binding, and in some cases, a spacer, due to its flexibility, can modify the system geometry and tune the interaction between the receiver and photoactive unit (Fig. 6).^36–39^ This recognition allows numerous electronic phenomena to occur; consequently, fluorescence “*turn-off*” or “*turn-on*” occurs.

Among the various analytes, many metals are considered harmful to tissues and organs after prolonged exposure. Diverse human health and environmental effects have been described in the literature; thus, the detection of heavy and transition metals is an essential goal in both the biological and ecological fields.^41^ Similarly, the synthesis of molecular sensors for the detection of anions is relevant due to their toxic effects in nature.^42^ For instance, CN^−^ is known for its extreme toxicity, and thus many conventional recognition approaches (*i.e.*, electrochemical, potentiometric, titrimetric, and voltametric methods) have been developed for its quantitative analysis.^43^ However, several of these methods use robust and expensive equipment, complex procedures and require a long analysis time.

### 1.3. Aims of this review

This contribution seeks to highlight some progress in the recent works (from 2016 to 2021) on fluorescent and colorimetric chemosensors (probes) based on NHCs for the recognition of highly toxic ions such as CN^−^ and Hg^2+^. Additionally, we present and evaluate the recognition units and fluorescent and sensing processes of these probes, providing valuable tools for designing new probes for the detection of hazardous ions. For the analysis and detection of ions, there is a plethora of information on sensors for different toxic species; however, the two above-mentioned species have a more significant environmental and human impact given that they can cause death in high concentrations. Likewise, it is vital to analyze and control the maximum limits of CN^−^ and Hg^2+^ to comply with current legislation to prevent some foods or residues in industry from harming human health.

This review focuses only on examples of NHCs without other heteroatoms (*i.e.*, O or S), increasing the importance of this group of compounds; in this case, up to two nitrogens are included in each ring. This review discusses the most recent advances in optical chemosensors for the detection of cyanide, and subsequently for mercury recognition. For both species, data will be presented on the LODs, absorption and emission bands, key further details, and association constants for complexes formed with Hg^2+^ ions. To the best of our knowledge, this is the first review that collects information regarding the application of N-heterocyclic compounds in the detection of highly toxic ions such as CN^−^ and Hg^2+^. Therefore, we hope that this review will be a helpful contribution to both applied heterocyclic synthesis and ion recognition, considering the high versatility of N-heterocycles.

## 2. Chemosensors for CN^−^ sensing

Cyanide is a highly toxic ion in nature and to humans. Its exposure at low concentrations (1.15 × 10^−4^ M) to humans *via* different routes such as inhalation, ingestion, and skin contact can cause chronic diseases and even death.^44^ In mammals, CN^−^ absorption causes cell death given that it inactivates mitochondrial cytochrome C oxidase, impeding the mitochondrial electron transfer cycle. Thus, CN^−^ inhibits oxidative phosphorylation and ATP production, leading to blockage of the cellular respiration process.^44–46^ Although cyanide is a deadly agent, many industries use it in mineral extraction, electroplating, and the manufacture of synthetic fibers. For example, illegal gold mining uses cyanide and mercury salts to extract the metal, poisoning the environment and the population that uses nearby water sources.

Due to the toxic scope of CN^−^, the World Health Organization (WHO) has established a limit for the concentration of this anion in drinking water of 1.90 × 10^−6^ M.^35,47^ Besides, cyanide has been used in different terrorist attacks worldwide,^48^ limiting the use of this raw material and establishing security measures to control its use. Therefore, in recent years, many researchers have focused their effort into developing economic and fast analytic methodologies where low LODs, high sensibility, and selectivity can be achieved. In addition, these methods must have an easy analytical preparation that can replace the conventional techniques such as gas chromatography (GC), GC-mass spectrometry (GC-MS),^49^ high-performance liquid chromatography (HPLC), and HPLC-MS.^50^ In this context, fluorescent and colorimetric probes have aroused the interest of many organic and analytical chemists given that they offer advantages in the above-mentioned situations.

**Fig. 7:**
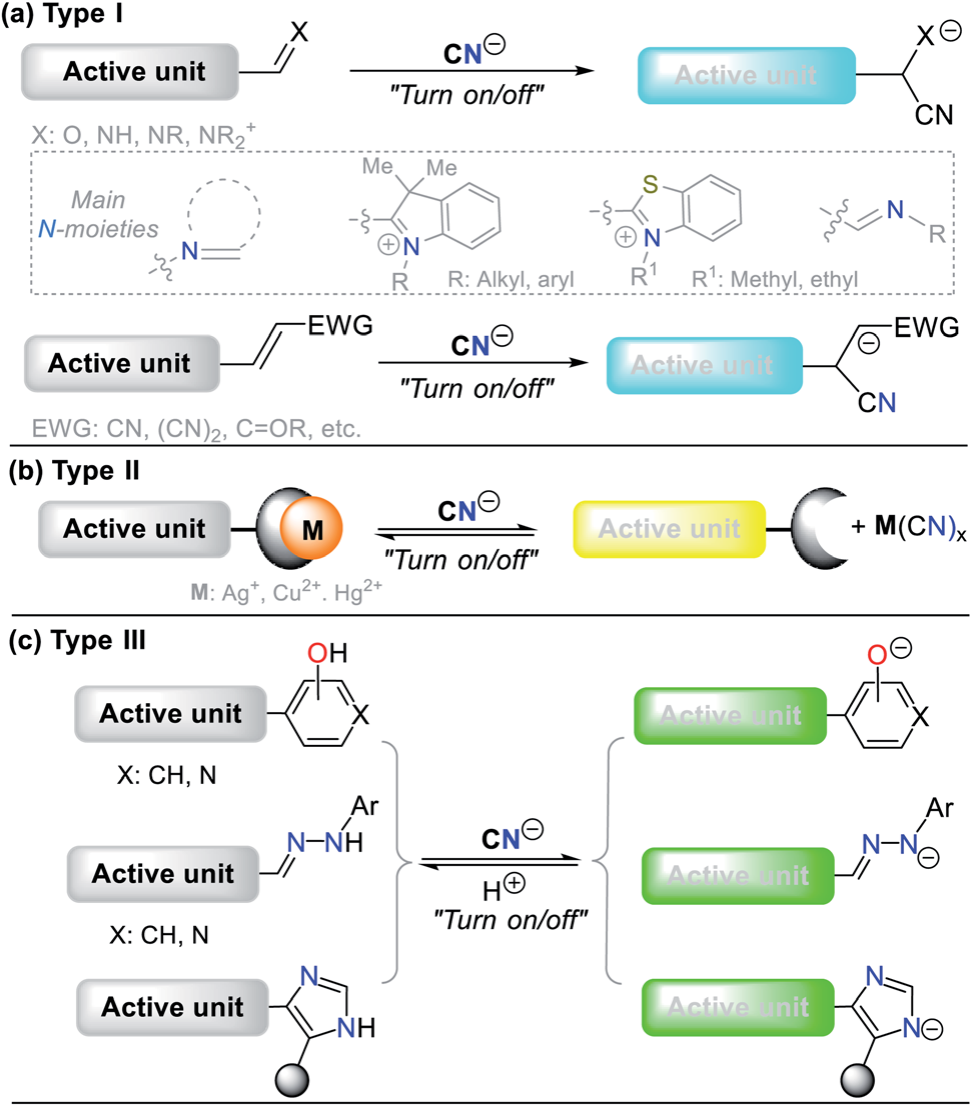
Probes for CN^−^ sensing. (a) Type **I**, (b) Type **II**, and (c) Type **III**.

The design of probes for sensing cyanide involves three different strategies. (**I**) Nucleophilic addition reaction of CN^−^ on an electrophilic carbon of a carbonyl (C=O), azomethine (C=N), or iminium (C=N^+^) group, and a carbon Cβ of a vinyl moiety conjugated with electron withdrawing groups (EWG) (Fig. 7a). (**II**) Displacement of a metal ion (often Cu^2+^) from the coordination sphere of a complex to yield a cyano-metallated compound (M(CN)_*x*_); in this case, the coordination sphere of the complex has a fluorophore, which being free from Cu^2+^, turns on the fluorescence (Fig. 7b). (**III**) The reaction of CN^−^ as a base to deprotonate the acidic hydrogen present in phenol derivatives (ArOH), hydrazones (R^1^HN–N=C–R^2^), or NH-heterocyclic compounds (Fig. 7c).

### 2.1. Pyrrole derivatives

Pyrrole derivatives are one of the most significant N-heterocyclic systems due to their excellent properties in different fields such as materials science, pharmacology, and polymers. Many pyrrole derivatives exist, among which some pyrroles, indoles, carbazoles, BODIPYs, and porphyrins stand out due to the high π-conjugation in these heteroaromatic–polycyclic systems.^50–56^ The pyrrole ring itself has rarely been applied as a molecular probe core in detection chemistry.

**Fig. 8:**
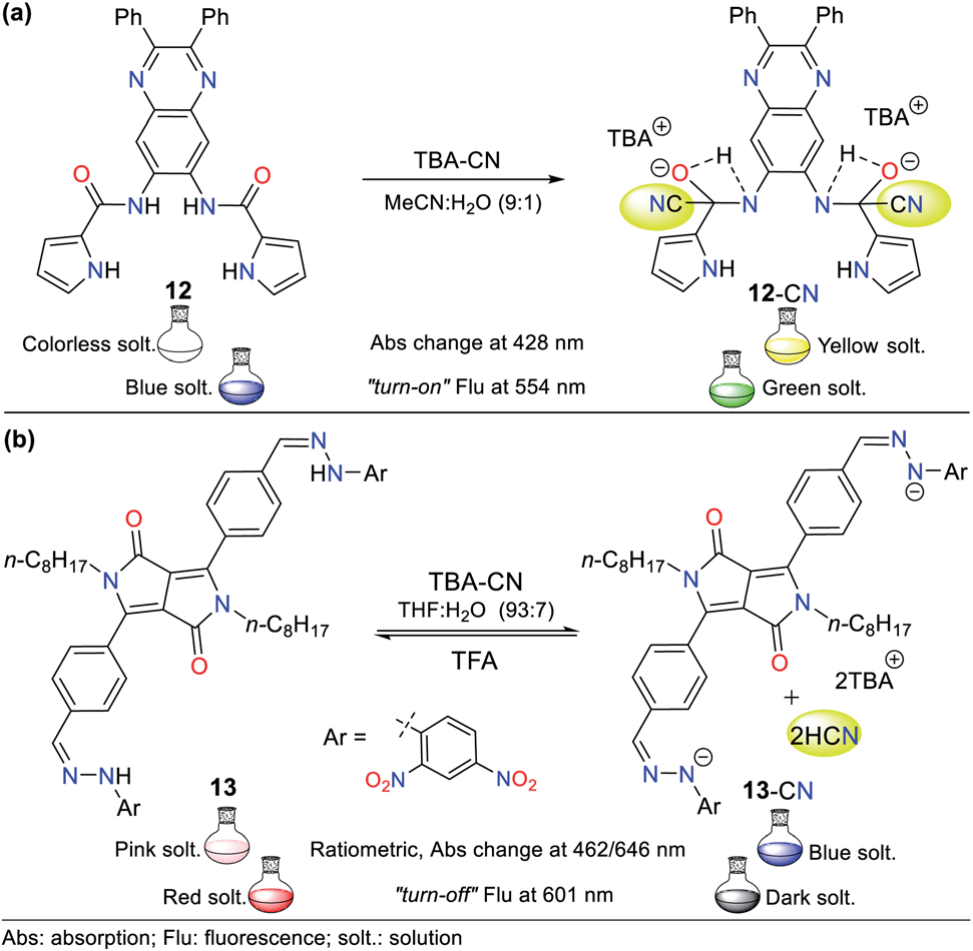
(a) Probes based on (a) pyrrole units 12 and (b) DPP unit 13.

Therefore, herein, we present only one example of a probe bearing a free pyrrole ring linked to a quinoxaline ring attached by an amide fragment (probe 12, Fig. 8a), which reported in 2006.^51^ This probe exhibits absorption bands at 291 and 378 nm and shows a weak emission band at 425 nm (*λ*_exc_ = 363 nm). After the addition of CN− to 12 in MeCN : H_2_O (9 : 1), the absorption bands decreased while three new bands appeared (at 299, 372, and 428 nm) accompanied with three isosbestic points (at 293, 360, and 391 nm), and a color change from colorless to yellow was observed. Likewise, the fluorescence studies showed that the emission band had a redshift with strong intensity at 554 nm, and the color of the solution changed from blue to green. This probe follows detection strategy I*via* the nucleophilic addition of CN− on the carbonyl groups (C=O) of the amide moieties in 12 (Fig. 8a).

In 2016, Wang *et al.*^52^ reported the synthesis of probe 13 based on a diketopyrrolopyrrole (DPP) bearing a hydrazone moiety as a selective colorimetric and “*turn-off*” fluorescent probe for the detection of CN^−^ (LOD = 2.30 × 10^−7^ M in THF : H_2_O). This probe works by detection strategy **III** with the DPP moiety as the fluorophore. The absorption spectrum of 13 showed two peaks at 396 and 516 nm. Upon the addition of CN^−^ to the solution, these two peaks shifted to 462 and 646 nm and were enhanced with an increase in the concentration of CN^−^, leading to the appearance of two isosbestic points at 315 and 439 nm. This variation was accompanied by a color change from pink to indigo. The emission studies showed that 13 was strongly fluorescent (*λ*_exc/em_ = 516/601 nm), which was quenched by up to 92% with the progressive addition of CN^−^, and a minor blue shift in its the emission band was observed. These results are attributed to the deprotonation of hydrazone, which enhances the electron-donating ability of the nitrogen atom, facilitating the PET process with fluorescence “*turn-off*” (Fig. 8b). Under a UV lamp, the probe solution appeared to fluoresce red and turn dark upon the addition of CN^−^.

#### 2.1.1. Indole derivates

**Fig. 9:**
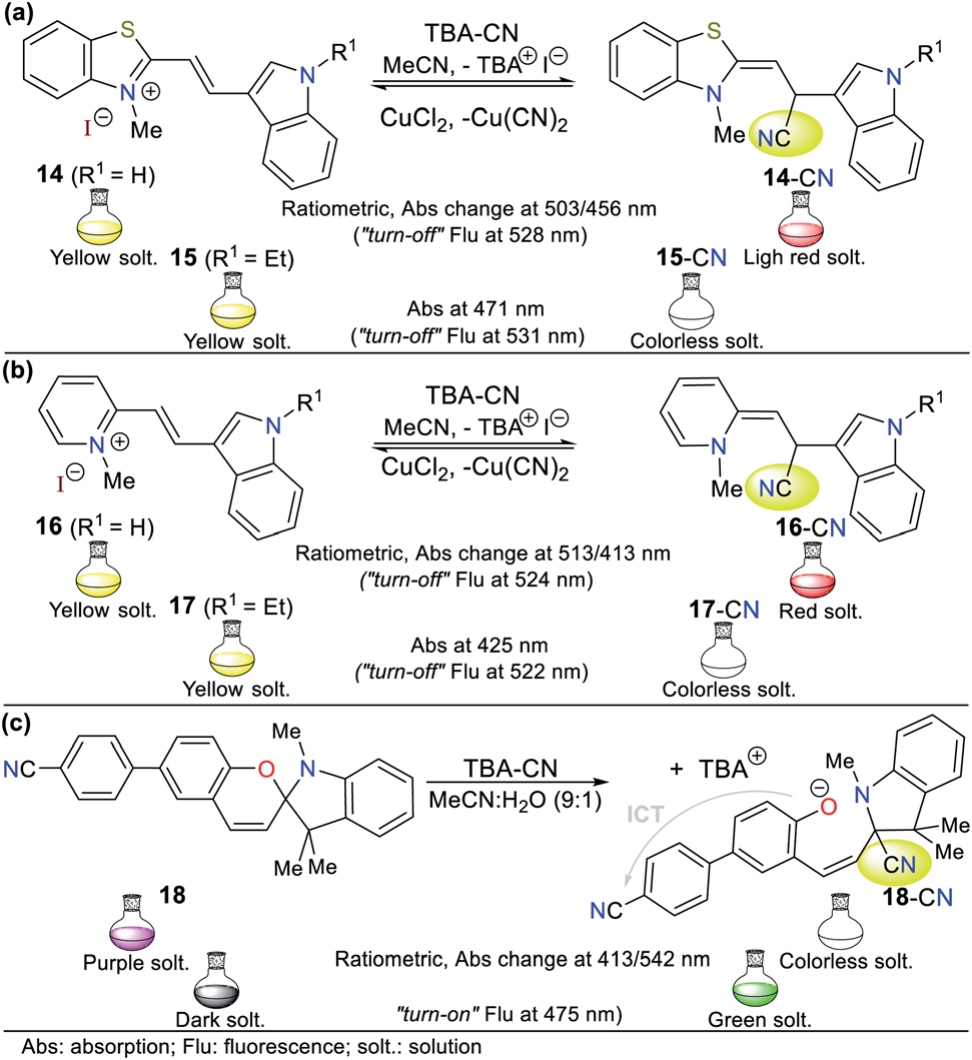
Probes based on indoles with (a) thiazolium salt 14/15, (b) pyridinium salt 16/17, and (c) spirochromene moiety 18.

In the case of benzo-fused rings, Wang and co-workers^53^ synthesized indole derivatives 14 to 16 bearing indolium and pyridinium salts as receptor units (Fig. 9). Compound 14 showed a strong absorption band at around 456 nm, which decreased upon the addition of CN^−^ and a new absorption peak appeared gradually at 503 nm, generating an isosbestic point at 467 nm and change in color from yellow to red. This probe showed yellow fluorescence at 528 nm, which gradually decreased with an increase in CN^−^ concentration (Fig. 9a). Similarly, 16 showed the main absorption band at 413 nm and upon the addition of CN^−^, the intensity of this absorption peak decreased, and a new peak appeared at 513 nm, generating an isosbestic point at 450 nm accompanied by a color change from yellow to red. This probe showed an emission band at 524 nm, which gradually decreased with the addition of cyanide, generating 30% fluorescence quench (Fig. 9b).

Alternatively, titration experiments with CN^−^ were carried out for compounds 15 and 17, showing absorption bands at 471 nm and 425 nm, respectively, which decreased with the addition CN^−^, and no new absorption bands were observed. Colorimetric changes from yellow to colorless and a gradual decrease in fluorescence were observed for both probes. In the sensing reaction for all the probes (14 to 17), the classic CN^−^ addition to the iminium group (C=N^+^) occurs; however, for 14 and 16, a hydrogen bond is also formed with the analyte followed by deprotonation of the indole ring NH group. Therefore, both strategies **I** and **III** are followed. After the addition of cyanide on the double bond, the intramolecular charge transfer is broken, thus quenching the fluorescence (Fig. 9a and b).

As the final example of indole derivatives, Erdemir and co-workers developed probe 18 this year.^54^ This probe showed a ratiometric absorbance change with bands at 415 and 542 nm. Once CN^−^ was added to 18 in MeCN : H_2_O (9 : 1), a new band emerged at 425 nm, and the color of the solution changed from purple to colorless. In its spectrum, the formation of an isosbestic point at 460 nm indicated the presence of an 18-CN adduct. Accordingly, the authors explored the emission properties of 18, revealing that the compound alone exhibit a weak emission band at 475 nm given that its ICT process is deficient. However, after the CN^−^ addition reaction on the spiro carbon atom of 18 (analogous to strategy **I**), an enhancement in fluorescence was observed due to the ring-opening of spiropyrane, making this probe a “*turn-on*” sensor for CN^−^ with an LOD of 2.08 × 10^−7^ M (Fig. 9c).

#### 2.1.2. Carbazole derivates

**Fig. 10:**
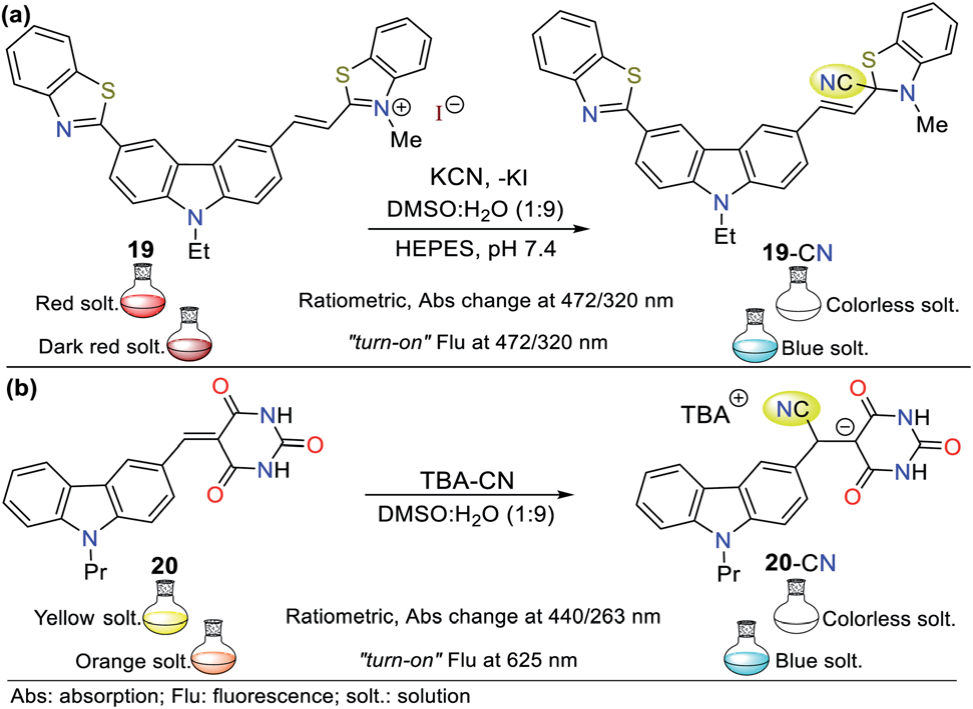
Probes based on carbazoles bearing (a) benzothiazole 19 and (b) barbituric acid 20 moieties.

The carbazole ring is a highly conjugated dibenzo-fused pyrrole with unique photophysical properties. Accordingly, Xueyi Sun *et al.*^55^ obtained derivative 19, which works as a colorimetric/fluorescent probe based on the ICT process (Fig. 10a). This probe shows an absorption band at around 320 nm attributed to π–π* transitions and a lower energy band at around 472 nm attributed to the ICT process. After the addition of CN^−^ to a solution of 19 in DMSO : H_2_O (1 : 9), the absorption band at 472 nm gradually decreased, while the band at 320 nm increased with an isosbestic point at 385 nm. A significant color change from orange-red to colorless was accompanied by this. This chemosensor follows strategy **I***via* the nucleophilic addition of CN^−^ to the C=N^+^ group in benzothiazole salt 19, inhibiting the π-conjugation and the ICT process. Similarly, two emission peaks were observed when probe 19 was excited at 330 nm (424 and 589 nm). The progressive addition of CN^−^ caused gradual fluorescence quenching of the band at 589 nm with an enhancement of the peak at 424 nm, generating an isoemissive point at 538 nm, which led to bright blue emission in the probe (LOD = 9.00 × 10^−8^ M).

Zou and co-workers reported another example in 2019,^56^ involving the synthesis of a carbazole-based colorimetric and fluorescent probe (compound 20) with AIE phenomena and LOD of 6.74 × 10^−8^ M. This probe exhibited absorption bands between 270 and 350 nm attributed to the π–π* transitions of the barbituric acid and the carbazole unit. Additionally, a sharp peak assigned to the ICT process from the carbazole ring to the barbituric acid moiety was observed at 434 nm. Upon the addition of CN^−^, the solution turned from yellow to colorless. Alternatively, fluorescence spectra were recorded in DMSO : H_2_O (1 : 9) given that water promotes the aggregation of the probe, leading to an enhanced emission. The studies showed that an increase in the content of water in the solution led to an AIE-active molecule with a strong fluorescence. When the mixture was H_2_O : DMSO (99 : 1), the emission peak appeared at 623 nm (*λ*_exc_ = 440 nm). Upon the addition of CN^−^, the fluorescence of the solution turned from orange to blue (Fig. 10b). In this case, probe 20 follows strategy **I**, meaning that cyanide acted as a nucleophile.

#### 2.1.3. BODIPY derivatives

**Fig. 11:**
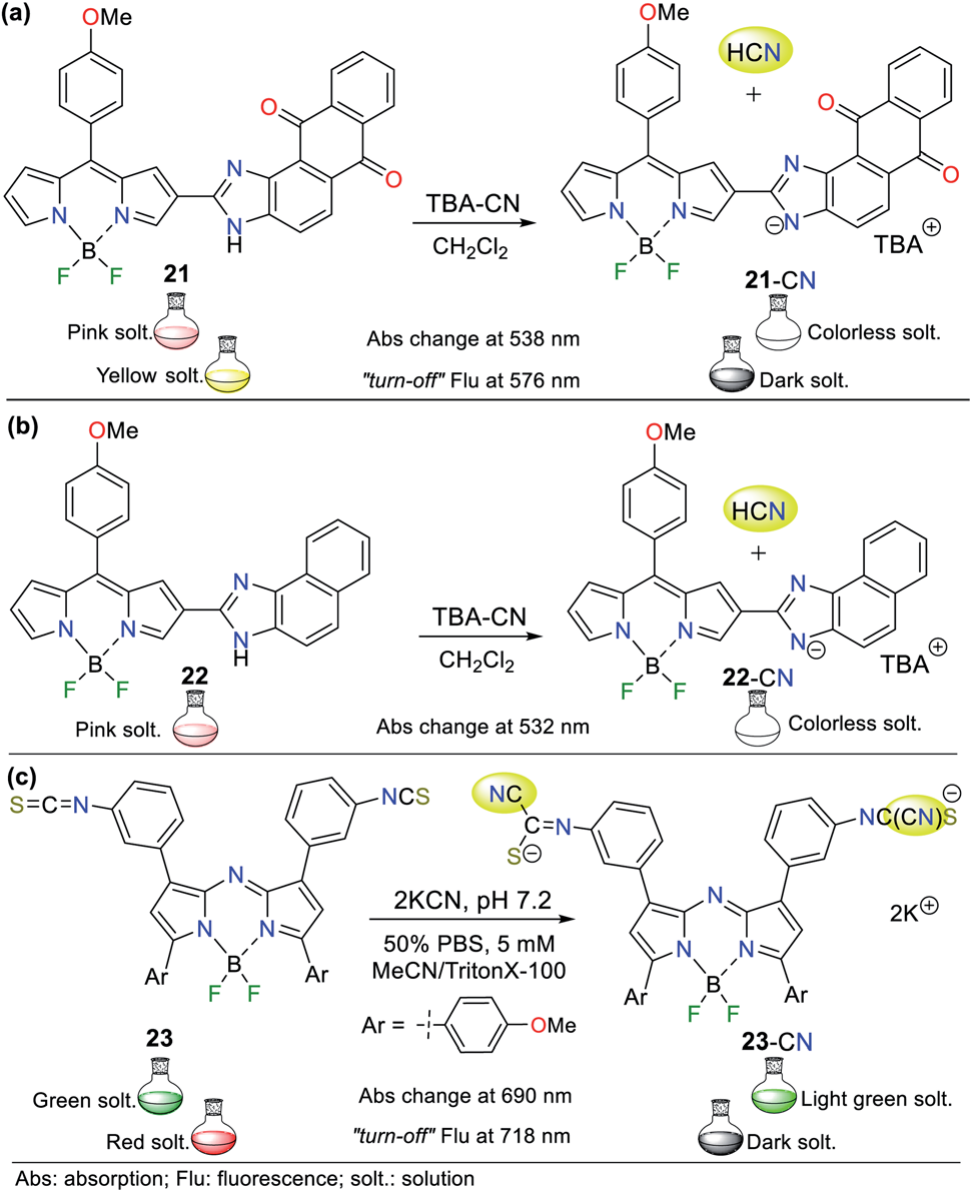
BODIPY derivates (a) 21, (b) 22 and (c) 23 for cyanide sensing.

BODIPY derivatives are a distinct type of fused pyrroles that are used as fluorescent probes because of the unique emission properties of their boron-dipyrromethene unit. In 2017, Dixit and Agarwal^49^ reported the synthesis of two imidazoaryl-BODIPY derivates 21 and 22 for cyanide sensing *via* strategy **III** (Fig. 11a and b, respectively). Both compounds showed absorption peaks in the high energy regions, which were assigned to n–π* (∼410 nm) and π–π* transitions (∼340 nm), while at around 535 nm, they showed an intense S_0_ → S_1_ (π–π*) absorption assigned to the BODIPY core. Studies showed that the solution turned colorless upon the addition of CN^−^, making these compounds suitable colorimetric probes. The fluorometric titration showed a decrease in emission intensity with an increase in CN^−^ concentration, leading to complete fluorescent quenching (band at 576 nm). It is important to note that the change in the emission properties was attributed to the N–H azolic interaction with the anion. Furthermore, the LOD was 1.09 × 10^−8^ M for 21 and 2.10 × 10^−4^ M for 22.

In 2019, Piyanuch *et al.* reported a fluorescent isothiocyanate-aza-BODIPY NIR (near infrared) probe for sensing CN^−^ with an LOD of 7.52 × 10^−7^ M (compound 23, Fig. 11c).^50^ This probe was tested in aqueous PBS buffer (pH 7.2) in acetonitrile (with TritonX-100), which showed fluorescence “*turn-off*” with the addition of the CN^−^ anion. Additionally, this molecule exhibited selectivity towards CN^−^ and discriminated other anions such as HPO_4_^−^, HSO_4_^−^, Br^−^, and NO_3_^−^. In this case, 23 works following design strategy **I***via* the nucleophilic addition of CN^−^ to the isothiocyanate moiety of 23, providing a more water-soluble dianion. The colorimetric studies showed that the absorption band at 690 nm decreased with the addition of CN^−^ accompanied by a color change from deep green to light green. Similarly, its emission spectrum showed a decrease in the band at 718 nm (*λ*_exc_ = 680 nm) with the addition of CN^−^, and a change in color from red to a dark solution under a UV lamp.

### 2.2. Pyrazole derivates

Pyrazole is a 5-membered heteroaromatic ring having two vicinal nitrogen atoms. Although few pyrazole derivates have been reported for the recognition of cyanide, our research group has interestingly advanced this matter. Among them, the most relevant compounds are those with a signaling unit based on fused systems such as pyrazolo[1,5-*a*]pyrimidines, and pyrazolo[3,4-*b*]pyridines. For example, our group previously studied probe 3, as shown in Fig. 1, which exhibited fluorescence “*turn-on*” by sensing mechanism **II**.

**Fig. 12:**
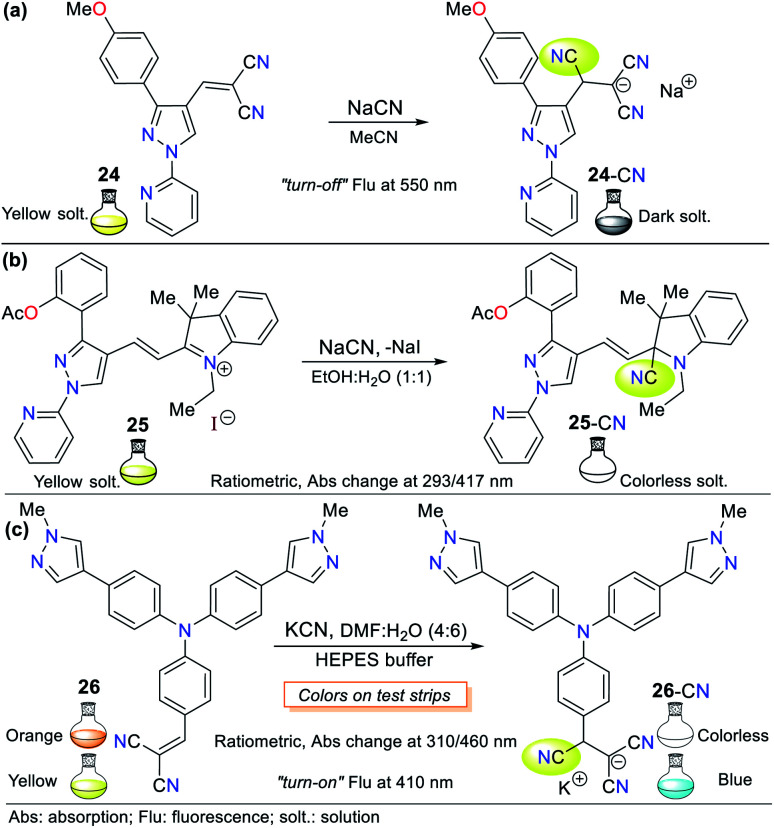
Probes based on functionalized pyrazoles (a) 24, (b) 25 and (c) 26.

Regarding the pyrazole ring itself, Orrego-Hernández and Portilla^57^ reported the synthesis of 3-aryl-4-dicyanovinyl-1-(2-pyridinyl)pyrazoles following design strategy **I**. Among the different aryl groups studied (4-O_2_NPh, Ph, and 4-MeOPh) in these pyrazoles, 4-methoxyphenyl derivative 24 is a fluorescent donor–π–acceptor (D–π–A) system that acts *via* the ICT phenomenon. This probe exhibited fluorescence quenching upon the addition of CN^−^ and presented a bigger absorption band (∼360 nm) compared to the other pyrazoles tested due to the donor character of its aryl group (Fig. 12a). Furthermore, this probe displayed high selectivity, appreciable quantum yields and a short CN^−^ sensing time with an LOD of 6.80 × 10^−6^ M. For 24, the ICT process is responsible for the high Stokes shift and bathochromic effect on its emission spectra with an increase in the solvent polarity.

Another pyrazole example based on probe 24 reported by us is the hemicyanine–arylpyrazole hybrid system, where the aryl group electron-donor character was modulated (*i.e.*, Ph, 4-MeOPh, 3,4,5-(MeO)_3_Ph, 4-AcOPh, and 2-AcOPh) to achieve a better design.^58^ 3-(2-Acetoxyphenyl)pyrazole 25 exhibited the best sensing properties but only as a colorimetric probe given that all the hybrid systems tested showed low fluorescence quantum yields in various solvents. This probe works through the nucleophilic attack of CN^−^ on its iminium group (C=N^+^), interrupting the D–π–A system. Consequently, a color change from deep yellow to colorless was observed in a solution of 25 (EtOH : H_2_O 1 : 1) with an LOD of 9.90 × 10^−7^ M (Fig. 12b).

The UV-vis spectra of probe 25 in solvents with different polarity exhibited two absorption bands (293/417 nm) attributed to the π–π* transitions and an S_0_ → ICT process from the pyrrole-like nitrogen (N1) atom in the pyrazole ring to the C=N^+^ group. The band at 417 nm disappeared upon the addition of CN^−^, indicating that the ICT process was broken, while the intensity of the other band increased for this colorimetric and ratiometric probe. Importantly, the steric effect occurring between the acetyl group and the azolic nitrogen (N2) in 25 prevents the π-conjugation from the aryl group towards the acceptor moiety; thus, ICT occurs only from N1, which possibly is the reason for its better performance compared to the other pyrazoles tested.^58^

Siva and Beneto reported a similar method using triphenylamine (TPA) derivative 26, having a dicyanovinylidene group as the receptor unit (Fig. 12c).^59^ Probe 26 also acts by design strategy **I**, where the addition of CN^−^ to the acceptor moiety interrupts the ICT process of the molecule, thus resulting in a suitable colorimetric and fluorimetric sensor. This probe exhibited two absorption bands at 310 and 460 nm, where the former increased with the addition of CN^−^, whereas the latter decreased, creating an isosbestic point and a change in color from red to colorless (LOD = 4.40 × 10^−8^ M). Similarly, fluorescence studies showed that 26 exhibits moderate blue fluorescence at 400 nm (*λ*_exc_ = 320 nm). The fluorescence intensity increased with the addition of CN^−^, turning the fluorescence of the solution to a stronger blue. Unfortunately, these findings were not quantified. Notably, the presence of a pyrazolic ring in 26 induces a bathochromic shift from around 440 to 460 nm in the absorption band in comparison to similar triphenylamine derivatives, which allows this compound to act as a ratiometric and colorimetric probe.

**Fig. 13:**
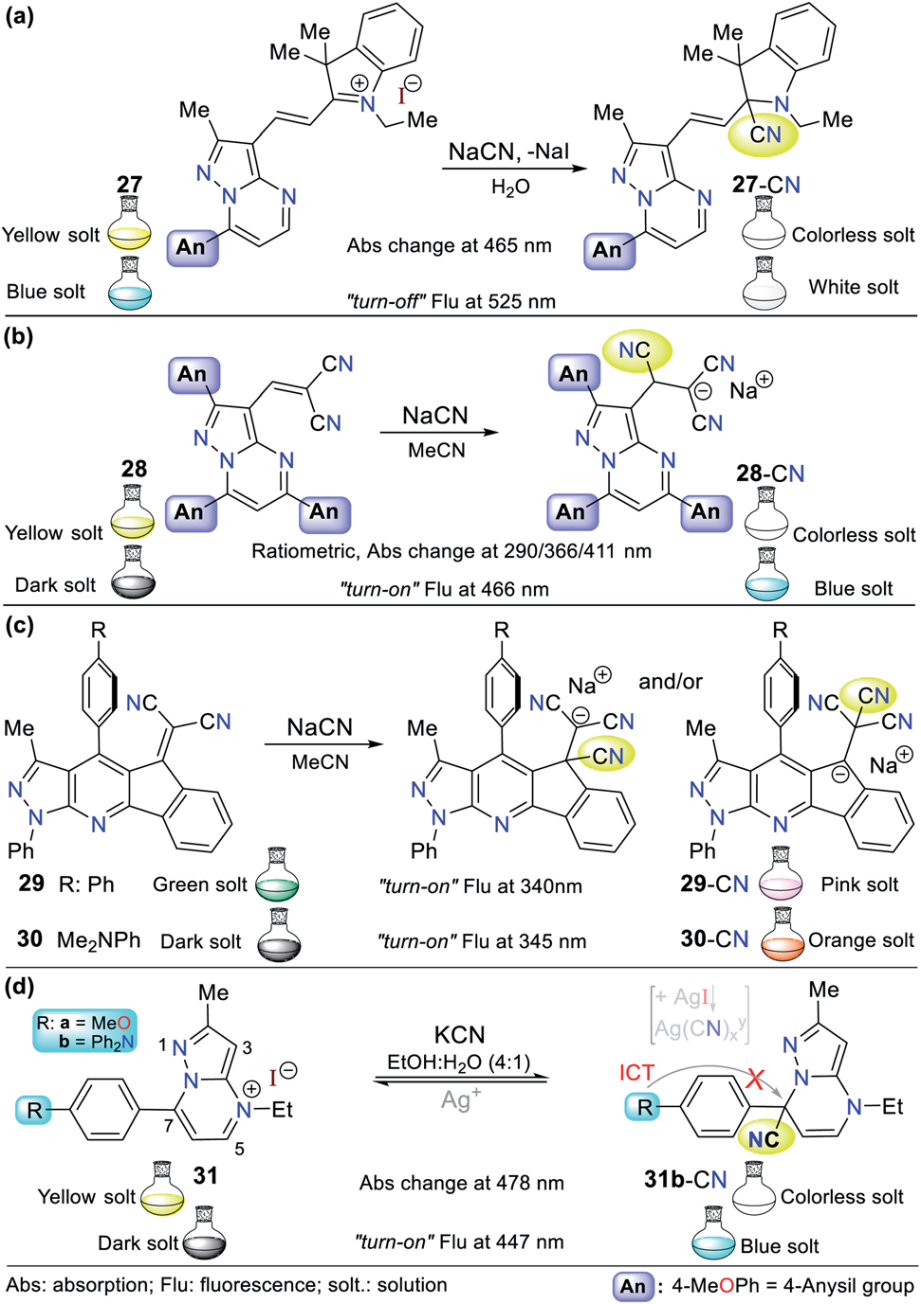
Probes having fused pyrazoles (a) 27, (b) 28, (c) 29/30, and (d) 31.

Alternatively, our group continued research on CN^−^ sensing, this time using fused pyrazoles, which evidently possess higher π-conjugation and structural rigidity than single pyrazoles, and thus enhanced photophysical properties. The first case is hybrid system 27 (a pyrazolo[1,5-*a*]pyrimidine–hemicyanine), which works as a colorimetric and fluorometric probe in 100% aqueous solution (Fig. 13a).^60^ The probe acts *via* the nucleophilic attack of CN^−^ on the C=N^+^ bond of its indolium moiety, which blocks the ICT process. This probe was tested on three different solvents (DCM, EtOH, and water) and showed a good quantum yield (*φ* = 0.42) in aqueous medium with an emission band at 525 nm (*λ*_exc_ = 464 nm). Upon the addition of CN^−^, color change was observed in solution from yellow to colorless (LOD = 6.00 × 10^−7^ M at 464 nm, *ε* = 41 400 M^−1^ cm^−1^), while the fluorimetric studies showed that the emission band was reduced accompanied by a color change from green-cyan to white (LOD = 8.60 × 10^−8^ M).

Inspired by the results obtained in the development of probes bearing pyrazole derivatives for cyanide sensing, we recently reported a second case based on the pyrazolo[1,5-*a*]pyrimidine (PP) core, but now connected to a dicyanovinylidene group.^61^ The effect of the peripheral 4-anisyl (4-MeOPh) substituents at positions 2, 5, and 7 on the fused ring was studied to achieve a better-designed probe. According to the results, probe 28 bearing three 4-anisyl groups exhibited the highest selectivity towards CN^−^ (Fig. 13b). Upon the addition of CN^−^ to 28 in THF : H_2_O (9 : 1), the three absorption bands (at 290/366/411 nm) generated varied, resulting in a change in the solution color from light yellow to white with an LOD of up to 6.50 × 10^−7^ M considering the three detection channels. The probe showed fluorescence “*turn-on*”, changing the color of the solution from yellow to light blue (*λ*_exc/em_ = 300/466 nm) with an LOD of 1.70 × 10^−7^ M. Both probes mentioned above (27 and 28) follow design strategy **I***via* the addition of CN^−^ on an electrophilic carbon. Notably, the PP core has been successfully used as a fluorochromophore linked with two types of recognition units (*i.e.*, dicyanovinylidene group and indolium salt) in the development of CN^−^ sensing probes.

A different fused pyrazole also reported by our group is based on the pyrazolo[3,4-*b*]pyridine core.^62^ In this case, some strategic products of an indeno[1,2-*b*]pyrazolo[4,3-*e*]pyridine-5-one library having aryl groups at position 4 of the fused-ring were used as precursors of the novel dicyanovinyl substituted systems. The products were found to be tunable ICT fluorophores and could be used as chemodosimeters for the detection of CN^−^. Two of the compounds tested, where the aryl substituents were benzene and 4-dimethylaniline (4-DMA) rings (29 and 30 in Fig. 13c, respectively), showed the best results.

The absorption spectra for probes 29 and 30 exhibited two characteristic bands at around 300 and 430 nm, which are attributed to the π–π* transitions and the S_o_ → ICT process from the pyrrole-like nitrogen of the pyrazole moiety to the nitrile groups. After the addition of CN^−^ to the probe solution in acetonitrile, the band at around 430 nm decreased, showing that the nucleophilic attack to the dicyanovinylidene moiety occurred. Thus, the sensor works by sensing strategy **I**. Curiously, the emission spectra of both compounds showed opposite results. Upon the addition of CN^−^, 29 (4-Ph, *λ*_exc/em_ = 340/490 nm) exhibited a new band at around 620 nm, whereas for 30 (4-DMA, *λ*_exc/em_ = 345/550 nm), its emission band decreased, which was accompanied by a color change from green to light pink and from dark to bright pink, respectively.

The last offered example about fused pyrazoles involves an ideal probe (compound 31b) also bearing a pyrazolo[1,5-*a*]pyrimidine core, in which Portilla and collaborators synthesized the novel pyrazolo[1,5-*a*]pyrimidinium salts 31a and 31b substituted at position 7 with anisyl (31a) or TPA (31b) as strong electron donor groups (EDGs), respectively.^63^ In this case, nucleophilic attack of the cyanide occurs on the N-heterocyclic core, which interrupts the π-conjugation, blocking the ICT process inside the probe molecule (strategy **I**). The absorbance experiments for 31b in EtOH : H_2_O (4 : 1) showed two bands at 327 (decreases) and 478 (increases) nm, which changed gradually with the successive addition of CN^−^ to the solution. The changes proceeded with the appearance of an isosbestic point at 370 nm and a color change in the solution from yellow to colorless. However, the fluorescence studies showed that the probe has a weak emission band at 447 nm, which increased with the addition of CN^−^, making this a “*turn-on*” probe.

In short, 31b detects CN^−^*via* notable selectivity/sensitivity (34 different species were tested) and three spectroscopic channels (LOD_(Abs)_ of 1.50 nM at 327/478 nm and LOD_(Flu)_ = 2.50 nM at 447 nm). Remarkably, 31b can act as a reversible probe by adding silver ions (from AgNO_3_). The sensing mechanism was confirmed using DFT calculations and ^1^H NMR and HRMS experiments, suggesting that the reaction proceeds *via* nucleophilic attack at position 7 of 31. Ultimately, the practical applicability of 31b was proven using test strips, studies in the solid state (with silica and almonds), and tap water.^63^

### 2.3. Imidazole derivates

**Fig. 14:**
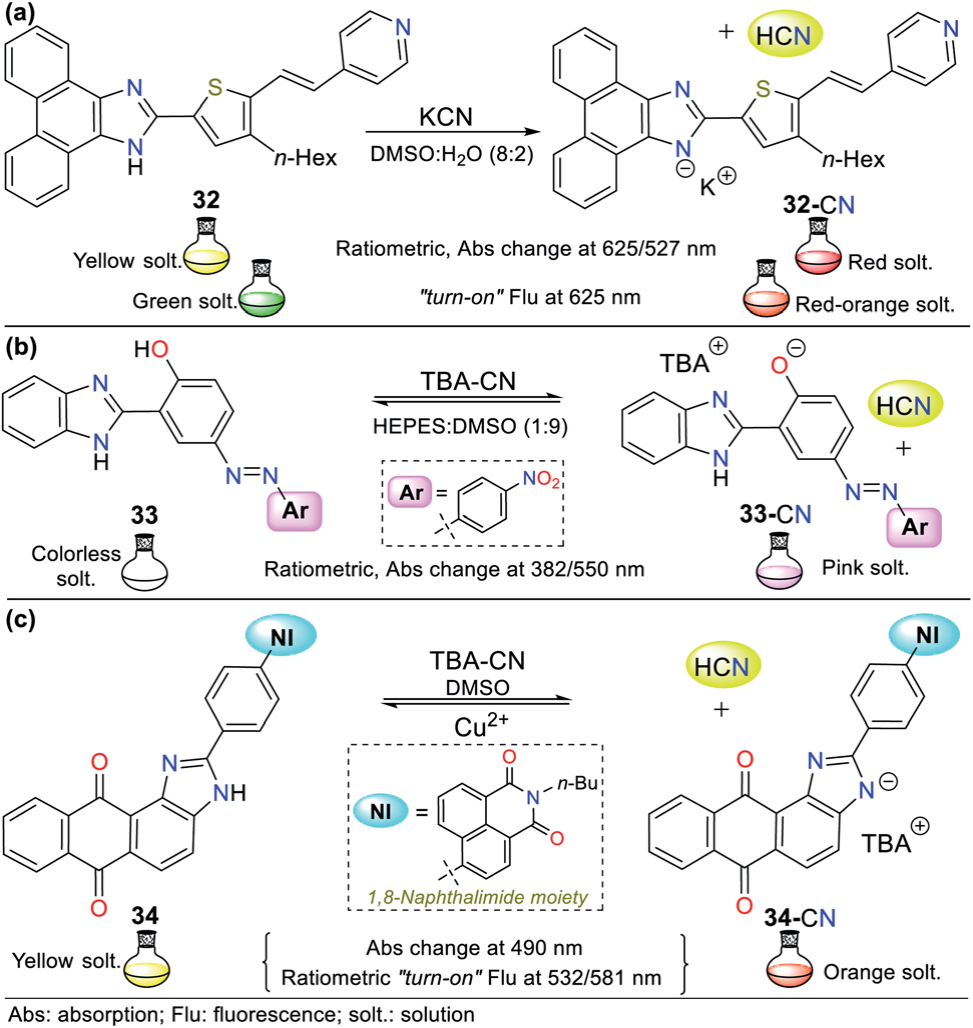
Probes based on fused *NH*-imidazoles (a) 32, (b) 33, and (c) 34.

Although the imidazole ring is usually found in natural molecules, few examples for sensing CN^−^ have been reported. Most probes are based on benzimidazole and other fused analogs. Accordingly, Siva and Beneto,^59^ in addition to pyrazole derivative 26 (see Fig. 12), synthesized phenanthro[9,10-*d*]imidazole 32 for the selective detection of CN^−^*via* design strategy **III** (Fig. 14a). This probe showed absorption (400 nm) and emission bands (527 nm) attributed to an ICT process from the fused-imidazole moiety to the pyridine ring of the substituent at position 2. After the addition of CN^−^ in DMSO : H_2_O (8 : 2), deprotonation of the NH-azolic occurs, improving the donor moiety electron density, and thus favoring the ICT process. The absorption spectrum of 32 showed three bands (at 300/400/460 nm), and upon the addition of CN^−^, the band at 400 nm was reduced while the two other peaks increased, producing two isosbestic points at 420 and 350 nm. The fluorescence studies showed that the emission bands at 527 and 625 nm gradually decreased and increased, respectively, with an increase in the concentration of CN^−^, generating an isoemissive point at 600 nm. The sensor changed its color from light yellow to red, which could be observed both by the naked eye and under a UV lamp, and the LOD was 3.30 × 10^−8^ M.

In an interesting example developed by Wang's research group, the authors synthesized and applied benzimidazole derivative 33 for the colorimetric recognition of CN^−^ with an LOD of 1.18 × 10^−9^ M in HEPES : DMSO (1 : 9, buffer solution).^64^ The absorption spectrum of 33 showed a moderate-intensity band at 382 nm due to the π–π* transitions and another band with weak intensity at 550 nm attributed to n–π* transitions. Firstly, competition studies were performed, demonstrating that with ratios lower than 1 : 9 of buffer solution, the probe was not only sensitive to CN^−^ but also to other anions such as F^−^, AcO^−^ and H_2_PO_4_^−^, although 33 showed the highest selectivity to CN^−^ in a 4 : 6 ratio of this solution. The addition of CN^−^ to the probe solution changed its color from colorless to pink, whereas no color change was observed with other anions (Fig. 14b).

Titration with CN^−^ revealed that the band at 382 nm in 33 decreased, while that at 550 nm increased, displaying ratiometric behavior with two isosbestic points at 366 and 448 nm. This probe works by sensing strategy **III**, where cyanide deprotonates the phenolic hydrogen of 33 and yields an anion that fortifies the ICT process from the oxygen to its nitro group. The imidazole–NH and phenolic–OH deprotonation were confirmed by ^1^H NMR and HRMS experiments, concluding that the reactions are not simultaneous given that OH acts first due to its higher acidity; therefore, an intramolecular hydrogen bond [N^*δ*−^⋯H^*δ*+^⋯O^*δ*−^] in compound 33 could be established (Fig. 14b).^64^

In 2018, Son and co-workers^65^ reported the synthesis of a sensor bearing anthra[1,2-*d*]imidazole-6,11-dione with a 1,8-naphthalimide moiety (34, Fig. 14c). In this case, the anion interacts with the N–H azolic group by deprotonation (strategy **III** for CN^−^ sensing). The colorimetric studies (in both solution and test strips) confirmed the CN^−^ selectivity of 34, showing a change in color from yellow to deep orange (under natural and UV light), which is attributed to the ICT process from the imidazole ring to the quinone fragment. Unfortunately, the solution also changed color to light orange in the presence of F^−^; thus, this sensor is not suitable for the detection of CN^−^ when fluoride ions are present as interferents in the sample to be analyzed. Emission spectra were recorded at two different excitation wavelengths (430 and 520 nm). Excitation at 430 nm showed an emission band at around 532 nm, and excitation at 520 nm exhibited a new intense peak at around 581 nm, in addition to the original peak at 532 nm. The fluorescence titration studies with CN^−^ showed ratiometric behavior at both excitation wavelengths. Additionally, the sensor was reversible in the presence of Cu^2+^ and suitable for working in the pH range of 2 to 10.

**Fig. 15:**
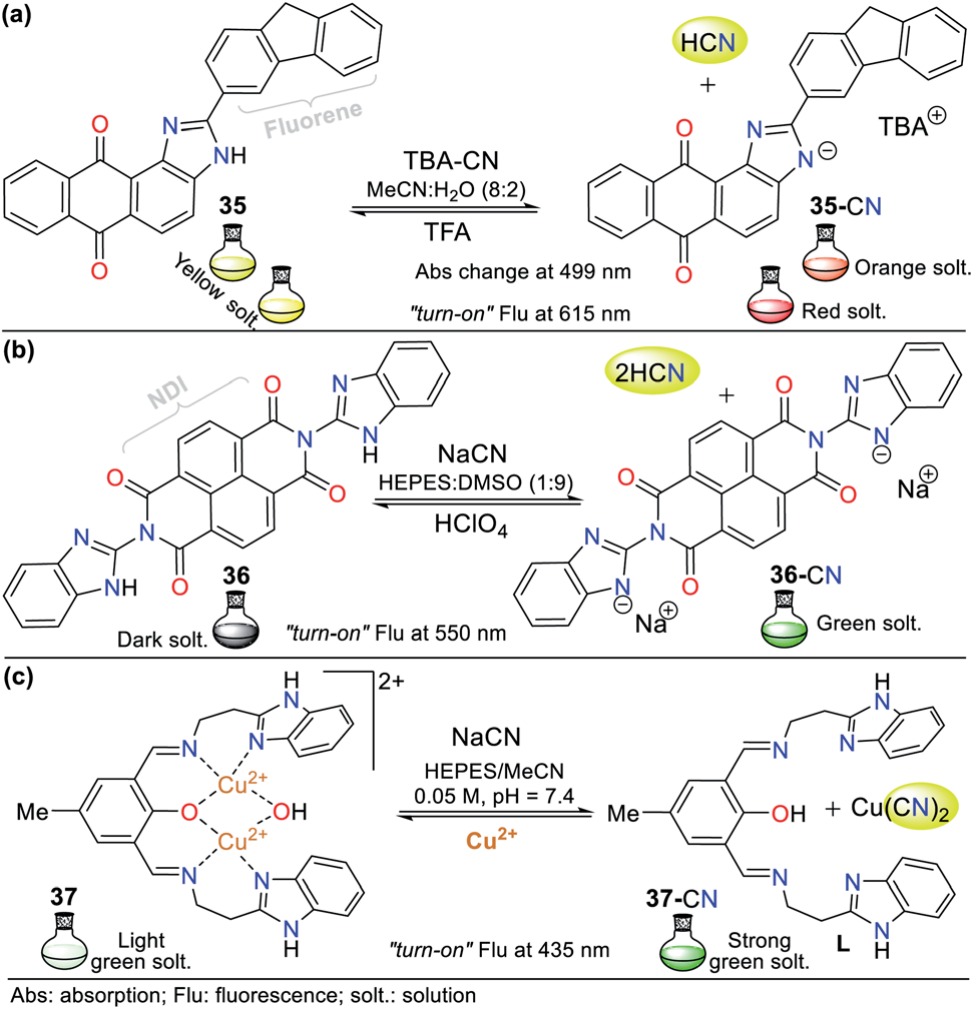
Chemosensors bearing fused imidazoles (a) 35, (b) 36, and (c) 37.

Recently, Bhaskar *et al.*^66^ obtained another anthra[1,2-*d*]imidazole-6,11-dione, but now conjugated to a fluorene moiety (35, Fig. 15a), which is capable of detecting CN^−^ in semi-aqueous media by both colorimetric and fluorimetric methods. The fluorescence studies showed that 35 itself exhibited a band at 585 nm (*λ*_exc_ = 415 nm), while the addition of CN^−^ to the solution led to quenching of the emission band together with a redshift (615 nm). Moreover, only in the presence of CN^−^, the solution turned from yellow to orange. Finally, the studies on pH within the range of 3 to 10 confirmed the suitability of the sensor for the analysis of real samples. This probe follows the fluorescence mechanism *via* the ESIPT process between the carbonyl group of the quinone moiety and the N–H azolic group. The addition of CN^−^ leads to the deprotonation of 35, interrupting the ESIPT process and quenching the fluorescence. Additionally, the absorbance studies showed two peaks at 415 and 499 nm, which decreased and increased, respectively, causing two isosbestic points at 455 and 359 nm. The absorbance and fluorescence LODs were found to be 5.30 × 10^−6^ M and 4.11 × 10^−8^ M, respectively.

Similarly, Lin and co-workers^67^ synthesized and studied the bis-benzimidazole 36 as a fluorescent probe for sensing cyanide in HEPES : DMSO buffer solution (1 : 9), which also has a naphthalene-tetracarboxylic diimide (NDI) fluorophore (Fig. 15b). The emission spectrum for 36 alone showed a maximum emission band at 510 nm (*λ*_exc_ = 455 nm), and when CN^−^ was added to the solution, the emission band shifted to 550 nm, with an enhancement in intensity. The color change in the sensor was from dark to green, and the LOD was found to be 8.32 × 10^−7^ M. In this case, CN^−^ deprotonates the benzimidazole hydrogen (N–H); therefore, the sensing strategy for probe 36 is **III**.

Recently, Anbu and co-workers^68^ reported an unusual probe (complex 37) compared to those described above where, the CN^−^ sensing mechanism occurs *via* addition or acid–base reactions. This new probe follows recognizing strategy type **II** and has the ability to act as a fluorogenic differential/sequential probe to identify Cu^2+^, Zn^2+^, CN^−^, P_2_O_7_^4−^ and DNA. The ligand (L) of complex 37 was obtained by reacting 2-(1*H*-benzo[*d*]imidazol-2-yl)ethanamine, 2,6-diformyl-*p*-cresol, and K_2_CO_3_ in ethanol at 40 °C. This ligand can chelate Cu^2+^ (37) and Zn^2+^ ions with a stoichiometry of 1 : 2 (L: Cu^2+^, LOD = 2.44 × 10^−8^ M) and 1 : 1 (L: Zn^2+^, LOD = 2.19 × 10^−9^ M), respectively. The presence of cations caused a decrease in the maximum absorption of L at 440 nm with a blue shift of around 18 nm due to the ligand–metal interactions. Similarly, the maximum absorption at 360 nm decreased markedly, and a new absorption band appeared at around 308 nm as a product of the charge transfer inside 37 and L: Zn^2+^ (Fig. 15c).

Complex 37 showed a broad band centered at 376 nm and three bands of lower intensity at 360, 385, and 405 nm. The fluorescence spectra of L showed an intense band at 515 nm, which decreased gradually with the addition of Cu^2+^ (2.50 × 10^−5^ M) and became red-shifted, resulting in a weak band at 530 nm. The addition of 5 equiv. of NaCN to 37 in MeCN/5.00 × 10^−4^ M HEPES buffer medium (pH = 7.4) generated a change in its emission spectrum. The intensity of the band at 530 nm increased and it was red-shifted by 5 nm, suggesting competition between L and CN^−^ by the Cu^2+^ ions. This competition restored up to 99.6% of the initial fluorescence of L and allows the use of 37 as a CN^−^ chemosensor with an LOD of 9.43 × 10^−9^ M. However, the mechanism by which L does not exhibit the same fluorescence spectrum once it has been released from Cu^2+^ is not clear. Nevertheless, this probe has high potential for the detection of CN^−^ in biological samples and other matrices such as different water sources.^68^

### 2.4. Pyridine derivates

Similar to the imidazole ring, the pyridine ring is very prevalent in the biological environment and studied in the development of probes for the detection of CN^−^. Accordingly, there are various pyridine derivates such as fused systems and organometallic or coordination complexes. Some crucial characteristics of pyridine derivatives, such as their electronic nature, basicity, synthetic versatility, high stability, and good optical properties, are the reason why these compounds are currently used in the design of chemosensors.^1–4,69,70^

#### 2.4.1. Pyridines

**Fig. 16:**
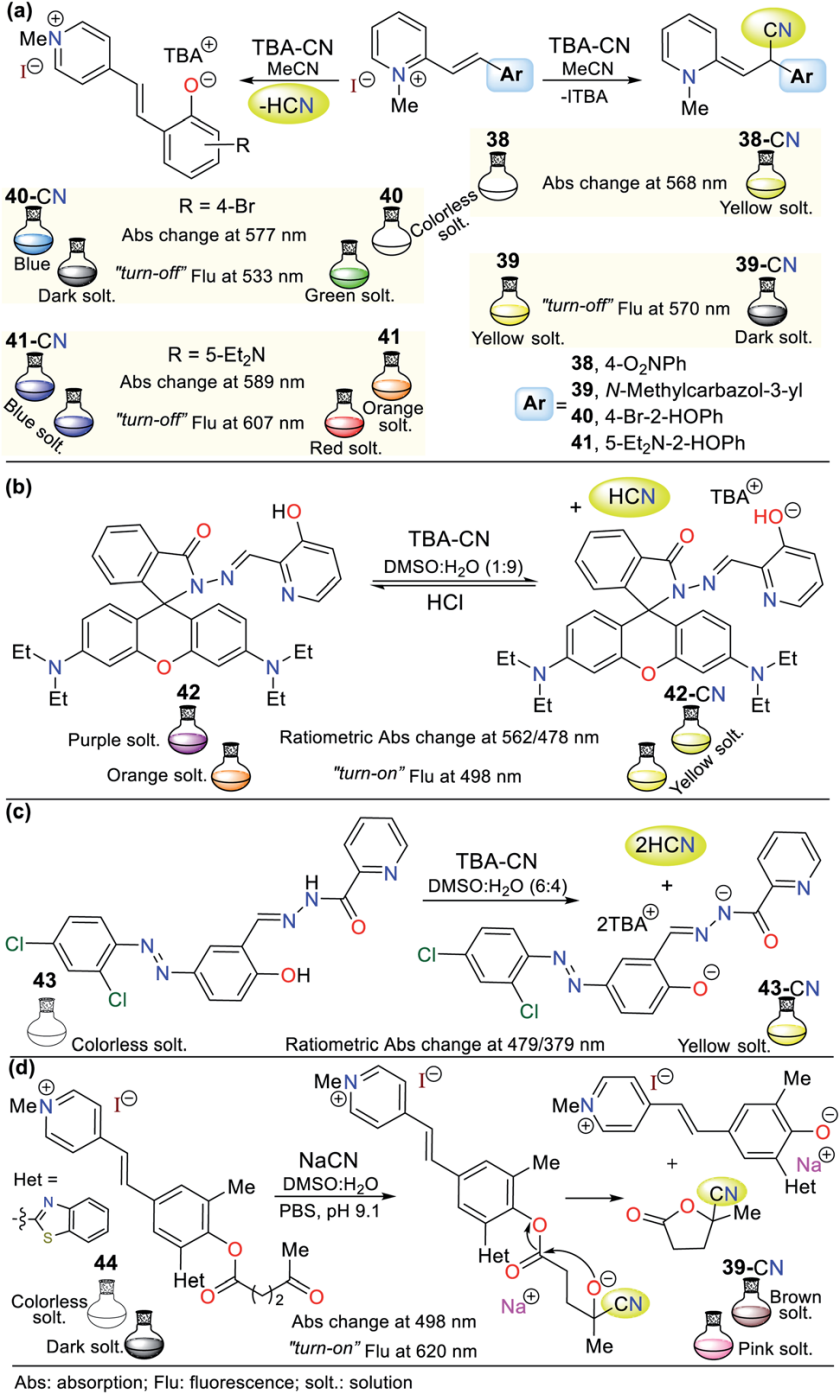
Probes having a pyridine ring (a) 38–41, (b) 42, (c) 43, and (d) 44.

Different CN^−^ probes based on hemicyanines with the structure of two aromatic rings linked by an ethylene group have been used, in which one of the rings bears an iminium group. Notably, some of these probes possess *N*-methylpyridinium salts in their receptor unit. In this regard, Yongxiao Xu *et al.*^71^ synthesized four dyes, which were proven to have different mechanisms of action for the detection of CN^−^ (probes 38 to 41, Fig. 16a). Probes 38 and 39 follow sensing strategy **I** by reacting with CN^−^*via* Michael nucleophilic addition on the ethylene group (C=C) activated with a pyridinium electron-withdrawing group (EWG). This mechanism was confirmed by ^1^H NMR studies, in which the typical signals of the ethylene group hydrogens disappeared. However, given that 40 and 41 have phenol rings, the acid–base reaction with the anion was confirmed *via* the ^1^H NMR signals of the ethylene group hydrogens; thus, the recognition unit for these probes follows strategy **II**, which acts by diminishing the fluorescence intensity during the sensing process.

In the absorption spectra of 38, the band at 336 nm decreased gradually with an increase in the concentration of CN^−^ and new peaks appeared at 425 nm and 568 nm. Additionally, an isosbestic point at 373 nm was formed. Similarly, the fluorescence intensity of 39 diminished by 11% with a blue shift of 6 nm from 570 nm to 564 nm. For probe 40, a fluorescence quenching was observed upon the addition of 1.4 equiv. of CN^−^ (band at 533 nm), while for analogous phenol derivative 41, only a 7% reduction in the intensity of the emission band was observed at 607 nm with a redshift of 21 nm. Possibly, the diethylamino group of 41 decreases its reactivity, and thus its sensitivity towards the cyanide addition reaction (Fig. 16a).^71^

Alternatively, Jing-Han Hu and co-workers synthesized a fluorometric sensor bearing 3-hydroxipyridine and rhodamine B moieties (probe 42), which follows strategy **II** of cyanide sensing by deprotonation of the OH group in 42.^72^ This was confirmed *via*^1^H NMR studies; in contrast, the OH group signal at 11.08 ppm disappeared after the successive CN^−^ addition. Similarly, the intensity of the aromatic signals weakened, which appeared at a high field. The color changes were evident to the naked eye, changing from colorless to yellow due to the addition of CN^−^. Furthermore, a variation in fluorescence from dark to shinny yellow for 42-CN was observed under a UV lamp, following the ICT fluorescence mechanism. Notably, compound 42 could be recovered upon the addition of HCl for up to 8 cycles without significant loss in its effectiveness (Fig. 16b).^71^

Around the same time, Zheng Li *et al.*^73^ synthesized a chromogenic probe (compound 43), in which the sensing route indicated that the cyanide deprotonates not only the OH group from the phenol ring but also the hydrazide hydrogen due to the highly basic character of this anion (Fig. 16c). The absorption spectra of 43 showed a band at 321 nm, which disappeared with an increase in the concentration of CN^−^ and exhibited new bands at 398 and 479 nm, allowing a color change from colorless to yellow to be observed in the presence of cyanide ions. The ^1^H NMR studies showed the disappearance of the signals at 12.64 (OH) and 12.07 (NH) ppm with the addition of 0.5 equiv. of CN^−^ and the Job method confirmed that the reaction stoichiometry was 1 : 2 (43-CN).

In another recent example, a hemicyanine pyridinium-based salt was used for CN^−^ sensing, which consists of probe 44 reported by Tang and co-workers.^74^ This probe has a levulinate moiety, and besides CN^−^, can detect hydrazine (N_2_H_4_) due to its unusual sensing mechanism (Fig. 16d). Moreover, 44 can obtained in a single step at room temperature, making it very attractive from an operational point of view; however, the access and cost of some of its precursors and poor reaction performance (31%) are considered significant drawbacks. The absorption spectrum of this compound does not exhibit any band between 400 to 600 nm. However, the addition of cyanide or hydrazine (N_2_H_4_) to a solution of 44 in DMSO : H_2_O (3 : 7) resulted in substantial changes in its absorption spectrum, with an intense band at 498 nm and a color change from colorless to brown, as shown in Fig. 16d.

Alternatively, its fluorescence spectrum shows a band at 620 nm, which increased with the gradually addition of CN^−^ or N_2_H_4_ with and LOD of 1.38 × 10^−6^ M and 5.47 × 10^−6^ M, respectively. These findings suggest a similar sensing process for both analytes *via* nucleophilic attack on the carbonyl of the ester group in 44 (strategy **I**). Then, intramolecular attack occurs, leading to fluorescent phenol derivative 44a and 3-carbonitrile-γ-lactone 44. The ^1^H NMR studies confirmed this sensing mechanism given that 44 has three signals located at high field assigned to the levulinate portion (CH_2_ × 2 and CH_3_), which disappeared upon the addition of CN^−^. In contrast, a new signal appeared at low field (∼12.60 ppm), which was assigned to the phenolic hydrogen. This probe was tested with real water samples, resulting in recovery rates of the probe for CN^−^ or N_2_H_4_ of 92–104% and 94–104%, respectively. Moreover, 44 was shown to be stable in the pH range of 9 to 11.^74^

#### 2.4.2. Quinoline derivates

Quinolines are benzo condensed pyridines, and given that their chemical nature is similar to that of pyridine, their N-heterocyclic moiety is electron deficient. Quinoline derivatives have fluorescent properties that can be increased by increasing the π-conjugation at position 2. Thus, some quinolines present crucial applications given that π-extended–systems favor π–π* and n–π* electronic transitions, and besides, their intrinsic photochemical stability makes them excellent candidates for use as fluorophores in the design of chemosensors.^1–4,69,70^

**Fig. 17:**
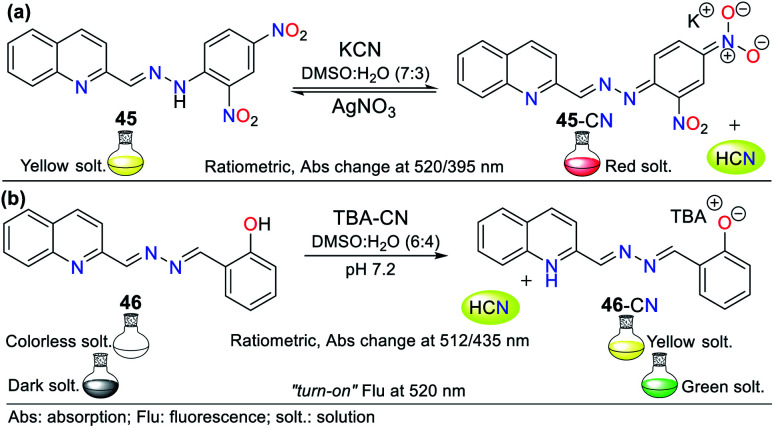
Probes for cyanide based on quinoline derivates (a) 45 and (b) 46.

This is the case of probe 45 designed by Wang *et al.*,^75^ which allowed the colorimetric detection of CN^−^ in semi-aqueous media. This probe works by strategy **III**, where the deprotonation of the hydrazone moiety produced an anionic species, leading to a redshift from 395 to 520 nm. This redshift happens given that the elimination of hydrogen in the hydrazone fragment increases the electronic density of nitrogen, reorganizing the charge of the molecule into canonic structures to finally obtain a stable delocalized structure. Thus, the proposed fluorescence mechanism for sensing CN^−^ with 45 is *via* the ICT process, which is accompanied by a color change from yellow to red. The UV-vis studies exhibited a decrease in the absorption band at 395 nm and an increase in the band at 520 nm upon the successive addition of CN^−^. The ^1^H NMR studies confirmed the presence of the anion, wherein the signal at 11.98 ppm is attributed to the disappearance of the hydrazone hydrogen. The addition of Ag^+^ to the solution of 45-CN reversed the sensing process (up to 4 reuse cycles), which was confirmed by ^1^H NMR studies with a similar spectrum to that of probe 45, with the signal at 11.98 ppm restored (Fig. 17a).

Another example following strategy **III** is that reported by Hu and co-workers^76^ in which the hydrazone of salicylaldehyde and quinoline 46 was synthesized. This probe showed a colorimetric response from colorless to yellow and a fluorescent response in the presence of cyanide (Fig. 17b). The colorimetric changes were studied *via* UV-vis spectroscopy and titration with 0 to 34.5 equiv. of CN^−^. Probe 46 showed a band at 320 nm, the intensity of which decreased with the addition of CN^−^, while the band at 435 nm increased, generating an isosbestic point at 385 nm. Similarly, the probe turned from dark to green under UV light, and the band in its fluorescence emission spectrum shifted from 440 to 516 nm with an increase in intensity upon the addition of 50 equiv. of CN^−^.

Both the colorimetric and fluorometric changes in 46 are attributed to the deprotonation of its OH group. This mechanism was confirmed *via*^1^H NMR studies, in which the OH group signal at 11.15 ppm disappeared when 2 equiv. of CN^−^ was added. Moreover, the aromatic signals exhibited high-field displacement. The action of CN^−^ on probe 46 resulted in delocalization of the charge in the conjugated system, resulting in the ICT process. Similar to the example mentioned before, this probe was reversible and could be used up to seven cycles in the presence of a protic acid. Finally, both probes (45 and 46) could be used in paper strips for the *in situ* detection of CN^−^ in aqueous samples.^76^

#### 2.4.3. Other fused pyridines

The synthetic versatility offered by NHCs is essential not only for introducing functional groups but for the construction of fused rings. Fused systems have proven to be of academic interest to study their chemical nature, reactivity, and structure–property relationship; likewise, their performance in the design new molecules with photophysical and biological properties is interesting from an industrial point of view. The molecular diversity in N-heterocycles offers a variety of applications. In CN^−^ sensing probes, fused pyridines or analogous compounds have been used to enhance the charge transport properties. Considering that pyridine has (4*n* + 2) π-electrons, it is aromatic, and thus an analog to benzene derivatives; similarly, quinoline is an analog to anthracene, a good fluorophore. In these systems, the delocalization of the charge and the nitrogen atom contributes to the formation of different resonance structures.^1–4,69,70^

**Fig. 18:**
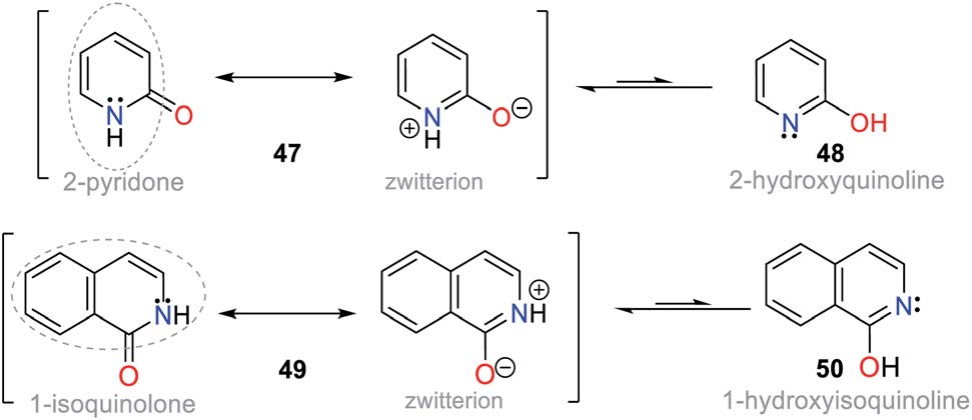
Tautomers of pyridin-2-ol 47/48 and isoquiolin-1-ol 49/50.

Accordingly, keto–enol tautomerism can result in two resonance structures for 2-hydroxypyridine (48). The keto form 47 corresponds to an amide and resonates with the enol structure (Fig. 18). Similarly, 1-hydroxyisoquinoline (50) is found as amide tautomer 49. This type of N-heterocycle system is used to promote the ICT process, where the formation of an anion produced by deprotonation in the presence of CN^−^ leads to charge displacement, and consequently different canonical structures are formed. In addition, strategically adding an electron donor group at specific positions of the ring can help compensate the electron deficiency, and thus favor the formation of a tautomer and stabilize the system.^1–4,69,70^

**Fig. 19:**
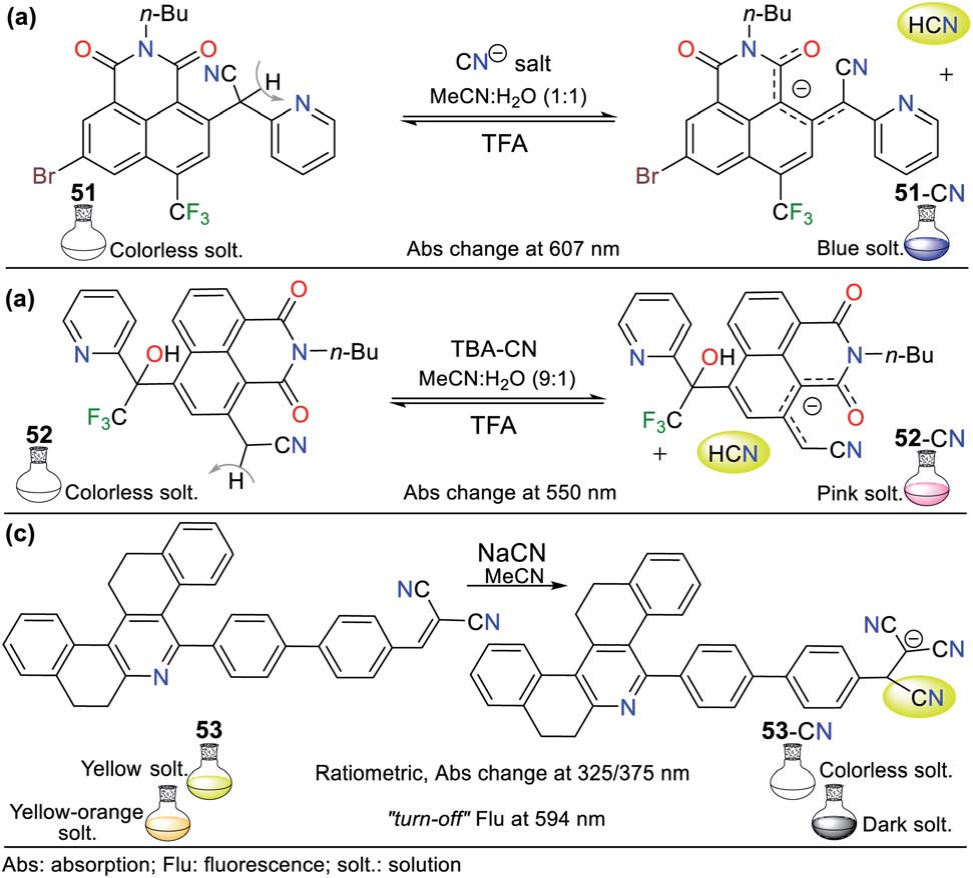
Probes based on fused pyridines (a) 51, (b) 52, and (c) 53.

Some fused pyridines for the selective detection of CN^−^ and exhibiting a resonance like 49 were studied by Liu and co-workers^77,78^ using the 1,8-naphthamide ring as the fluorophore for ICT processes. Initially, the authors introduced a 2-(pyridin-2-yl)acetonitrile group at position 2, obtaining probe 51. For this, they blocked position 6, avoiding the formation of isomers.^77^ This probe was evaluated against different ions, proving to be active for CN^−^. Upon the addition of the anion in MeCN : H_2_O (1 : 1), the absorbance band at 607 nm increased, causing a change in the color of the solution from colorless to blue. This optical result is due to the deprotonation of one of the Hα adjacent to the nitrile group. The stability of the carbanion formed is favored by the resonant effects, similar to that mentioned above. However, some ions such as HSO_4_^−^, F^−^, H_2_PO_4_^−^, and AcO^−^ were interferents given that they also caused color changes (Fig. 19a).

Next, the authors reported the synthesis of another 1,8-naphthamide derivative (probe 52). It was not necessary to block position 6 of the N-heterocyclic moiety because the substituent at position 4 favors cyanomethylation at position 2 (Fig. 19b).^78^ The solution of 52 in MeCN : H_2_O (9 : 1) was colorless. However, an increase in the absorbance band at 545 nm occurred upon the addition of CN^−^ to the solution, resulting in a change in color to a pink hue. Unfortunately, the presence of S_2_^−^, HSO_4_^−^, F^−^, and AcO^−^ was shown to be interfering ions. Unlike the other probes that follow design strategy **II**, this probe offers an advantage by deprotonating a Cα instead of groups such as phenols or amines, and thus notable changes in stock displacements are obtained.

Notably, the reversibility of probes 51 and 52 was evidenced by using a proton source such as trifluoroacetic acid (TFA), allowing these probes to be used for several cycles. Probes 51 and 52 achieved LODs of 1.22 × 10^−5^ M and 5.53 × 10^−7^ M, respectively; thus, only probe 52 can detect lower levels of CN^−^ than that allowed by the WHO. Despite this, the works carried out by Liu and co-workers are a clear example of chemosensor design for the detection of anions.^78^

In this case, Manickam and Iyer^79^ developed probe 53 for sensing CN^−^, having tetrahydrodibenzo[*a*,*i*]phenanthridine. This probe showed a color change from yellow to colorless with the addition of CN^−^ in acetonitrile according to sensing strategy **I** (Fig. 19c). The UV-vis studies showed a hypsochromic effect in the absorption band at 375 nm, changing to 325 nm, with an isosbestic point at 338 nm. Similarly, when evaluating the fluorescence of 53 under a UV lamp at 365 nm, the probe changed from yellow-orange to dark. The fluorescence spectrum of 53 showed a band at 594 nm, which decreased in intensity upon the addition of CN^−^. The sensing mechanism of 53 involves the resonance effect from the pyridine ring through biphenyls.

The absorption spectrum of probe 53 shows three bands, an intense at 375 nm and two lower intensity bands at 270 nm and 298 nm, evidencing ICT processes due to its high π-conjugation.^79^ This probe also was evaluated by cyclic voltammetry, proving that the addition of CN^−^ causes the reduction potential of 53 at −1.41 eV to shift towards more negative potentials, reaching up to −1.07 eV for the adduct 53-CN. These results show that the sensing mechanism involves a shutdown by the ICT process from the N-heterocyclic core to the dicyanovinylidene moiety in 53. Finally, this probe achieved a better LOD than that reported by Liu *et al.*,^77,78^ reaching 3.93 × 10^−8^ M with a rapid response of around 20 s in a wide pH range of 3 to 11.

### 2.5. Metal complexes

**Fig. 20:**
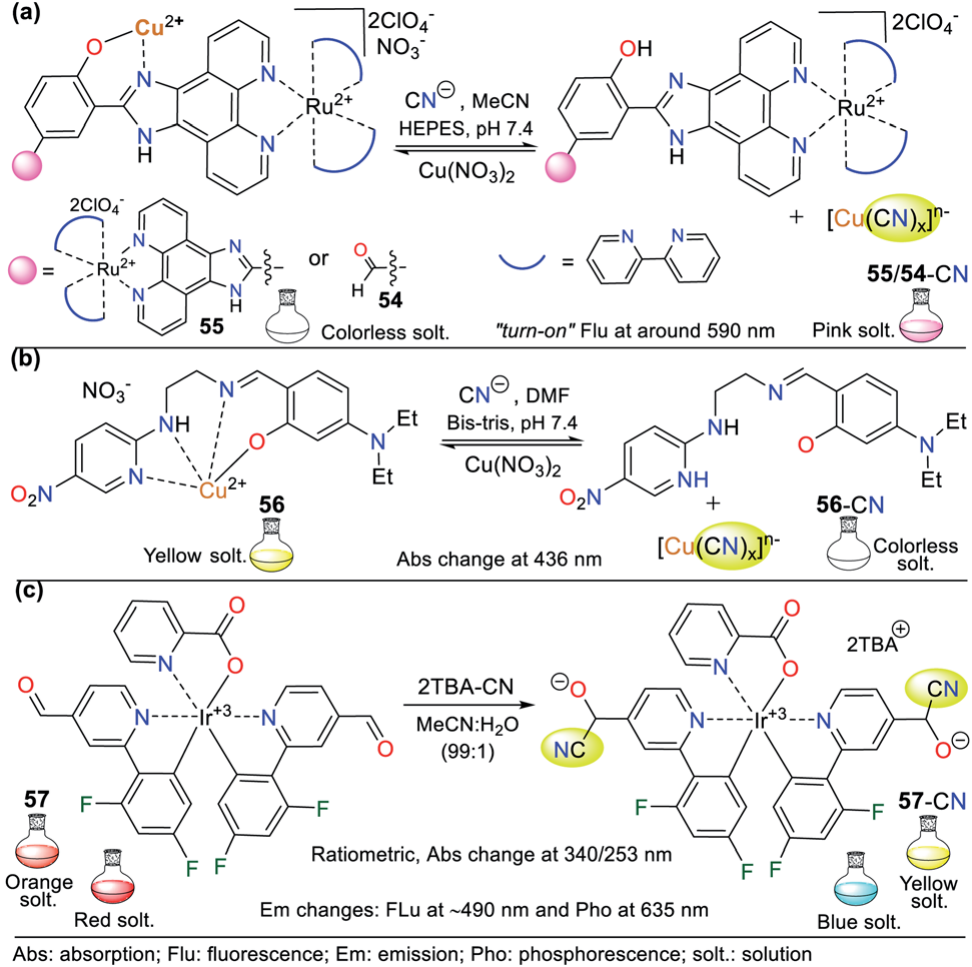
Probes for sensing CN^−^ based on coordination compounds bearing (a) ruthenium, (b) copper, and (c) iridium.

Various NHCs have been proven to be functional scaffolds for developing chemosensors. For example, some pyridine derivatives are part of coordination compounds with ruthenium(ii) and iridium(iii), which have the following photophysical advantages with respect to organic fluorophores: large Stokes displacements,^80^ high stability,^81^ relatively long emission lifetimes, and emission wavelengths in the visible region.^82^ In these probes, the coordination complex is the signaling unit, and the recognition moiety is generally an electron-deficient carbon; thus, various probes have been applied. For example, Zheng *et al.* developed probes 54 and 55, where an Ru(ii) complex moiety is the signaling unit, and another complex of Cu(ii) is the recognition site (Fig. 20a).^83^ These probes have very weak emission bands at around 590 nm, but without Cu^2+^, the intensity of their bands increase dramatically. Consequently, these probes were used for sensing CN^−^ following strategy **II**, evidencing high sensibility even in the presence of chelating agents such as EDTA due to the high affinity of CN^−^ for Cu^2+^. The LOD for the probes was 3.60 × 10^−7^ M for 54 and 8.70 × 10^−7^ M for 55. Besides, they were not selective towards other anions that can interact with Cu^2+^.

Pyridine derivatives are not only used to facilitate the resonance effects in ICT phenomena or as EWG to increase the electrophilic character of vicinal carbons to the ring. They have also been used to create coordination bonds with metallic ions, taking advantage of the free electron pair on the nitrogen atom. In particular, copper complexes are used to detect CN^−^ due to the high affinity of this metal for the CN^−^ anion. In the detection process, a stable complex of [Cu(CN)_*x*_]^*n*−^ is formed together with a free ligand, which was evidenced due to the variations in the photophysical properties of the sensor solution.^83,84^

For example, Kang *et al.*^84^ studied the Cu^2+^ complex 56, which was formed *in situ* from a ligand bearing two aromatic rings (the EWG 5-nitropyridin-2-yl and the electron-releasing group (ERG) dimethylaminophenol), both connected by an imine bridge with ethylenediamine. Regarding the probe design, the ligand molecule was colorless in solution and allowed the colorimetric detection of Cu^2+^ (LOD = 8.80 × 10^−7^ M) by a color change to intense yellow and the formation of 56. The ligand molecule became free again by adding CN^−^ to a solution of complex 56, as evidenced by the color change from yellow to colorless. The absorption spectrum of 56 showed a band at 436 nm, which diminished after the gradual addition of CN^−^ (LOD = 2.72 × 10^−5^ M) (Fig. 20b).

Organometallic compounds of 2-phenylpyridine derivatives have also been used as signaling units in probes to detect cyanide, in which the emission is due to phosphorescence. Accordingly, Reddy and co-workers synthesized chemodosimeter 57, a complex bis[[2′,6′-difluorophenyl-4-formylpyridinate]-*N*,C4′]iridium(iii). The detection occurs with the formation of cyanohydrin following strategy **I** of the probe design and *via* the nucleophilic attack of CN^−^ on the formyl group of 57 (Fig. 20c).^85^ The emission spectrum of 57 in acetonitrile showed a band at 635 nm, while in the presence of 2 equiv. of CN^−^, it shifted to 480 nm with a color change from red to blue. Moreover, the intensity was enhanced by 536 times, giving a quantum yield of 11%.

The formed adduct 57-CN was confirmed to have a 1 : 2 stoichiometry with high selectivity towards the analyte and an LOD of 2.16 × 10^−8^ M. The ^1^H NMR experiments revealed that 57 has a signal at 10.17 ppm (s, 2H), which is assigned to its formyl groups. This signal decreased with an increase in the concentration of CN^−^, while a signal appeared at 7.57 ppm, evidencing the formation of cyanohydrin. The disappearance of the aldehyde signal was evidenced when the reaction was completed upon the addition of two equiv. of the analyte. The FTIR spectrum of the probe showed the disappearance of the C=O stretching band at 1706 cm^−1^ and the appearance of the C≡N band at 2248 cm^−1^ upon the addition of cyanide.^85^

**Fig. 21:**
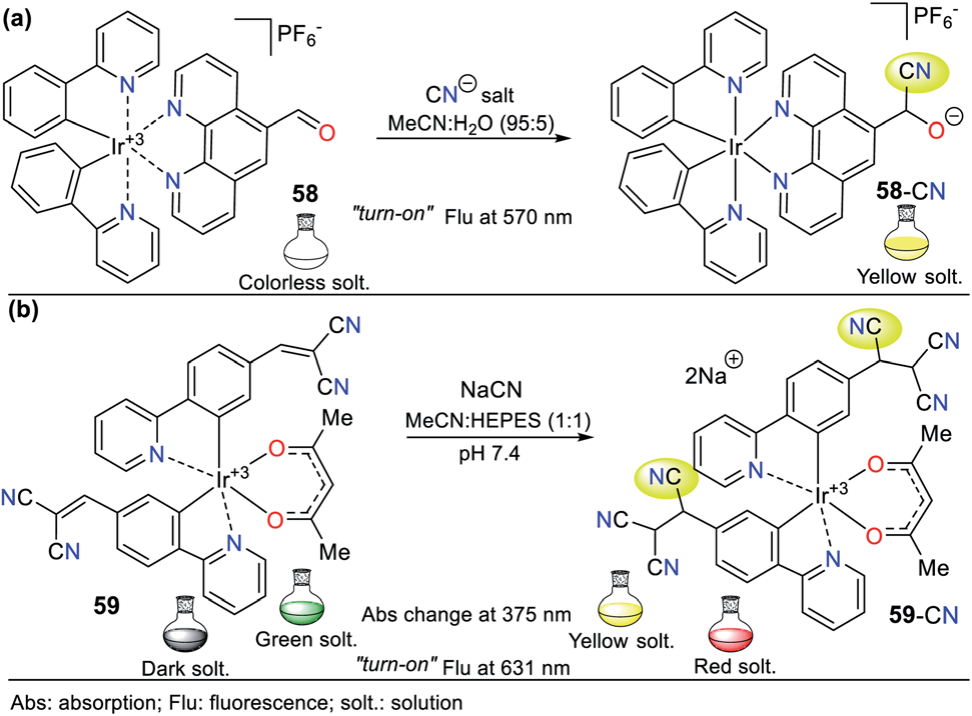
Probes for CN^−^ based on iridium complexes (a) 58 and (b) 59.

Recently, Lin and colleagues obtained probe 58, which also consists of an iridium(iii) complex formed with 2-phenylpyridine ligands, and in this case, they used 5-formyl-1,10-phenantroline as an auxiliary ligand (Fig. 21a).^86^ Similar to probe 57 designed by the Reddy group,^85^ the formyl group of 58 acts as the recognition unit through the cyanohydrin 58-CN. The solvents used in this type of probe play a crucial role in the analytical signal intensity, where the most widely used mixture is MeCN : H_2_O. In this case, a 95 : 5 ratio was used, which showed no variation in the intensity of the emission bands. Upon the addition of cyanide, the absorption spectrum of 58 exhibited two bands at 267 and 380 nm, which are attributed to the metal–ligand charge transfer (MLCT) process. This behavior is typical in metalorganic complexes using carbonyl groups or Michael acceptors as recognition units. The color of probe 58 changed from light yellow to shiny orange under a UV lamp (365 nm) upon the addition of CN^−^. Even though its recognition unit has only 1 equiv. to detect CN^−^, its emission spectrum showed an enhancement of up to 15 times in the intensity of the band at 570 nm, giving a quantum yield of 28%.

Alternatively, the fluorescence intensity of 58 became stable at 130 s when it reacted with 1 equiv. of CN^−^. Similar to the above-mentioned example, the high specificity towards the formation of cyanohydrin makes this probe selective to CN^−^ in the presence of 17 other anions. This process was studied *via*^1^H NMR, applying the same method as that for 57, where CN^−^ was successively added and analyzed. The spectra showed a diminution in the signal at 10.56 ppm, corresponding to the formyl group, and a progressive increase in the signal at 8.6 ppm, which is assigned to the formed cyanohydrin. In these types of complexes, density functional theory (DFT) studies display that the HOMO is localized mainly on the metal center, while the LUMO is localized in the zones in which there is a lower electronic density. For 57 and 58, the LUMO is localized in the aldehyde.^85^

A different approach regarding the recognizing unit for this type of probe was developed by Kim *et al.*, who developed probe 59, which combines electrochemiluminescence with sensing CN^−^.^87^ For the sensing process, 59 has a dicyanovinylidene group, working *via* strategy **I**. This probe exhibited poor CN^−^ selectivity because sulfide ions (S^2−^) and thiols such as cysteine are interferent species, which could form similar adducts to 59-CN (Fig. 21b). However, under an oxidation potential of 1.2 V, 59-CN showed unique photophysical properties including luminescence at 631 nm, which could be observed by the naked eye; in contrast, the adducts formed with the interferents only had weak luminescence at 1.5 V. Consequently, the electrochemical manipulation towards the positive potentials of the probe allowed it to have a detection limit of 4.00 × 10^−8^ M for cyanide. Additionally, DFT calculations demonstrated that the formed adduct 59-CN has a higher HOMO than that formed with S2^−^ and cysteine, making it more susceptible to oxidation at a lower potential, thus discriminating between these interferents.

## 3. Chemosensors for Hg^2+^ sensing

Similar to cyanide, mercury is a hazardous toxicant raw material that impacts human health and the environment. The concentration and speciation of mercury in the atmosphere depend on the proximity to its sources, the availability of oxidants, the concentrations and properties of aerosols, regional and global-scale meteorology, and surface conditions.^88^ Mercury deposited in the terrestrial environment can cause environmental harm given that it is transported to aquatic systems, where it can be methylated and then bio-accumulated in the aquatic food chain. Human or animal consumption of high trophic-level fish or other foods that have been contaminated with mercury can lead to toxic effects.^89^ The United States Environmental Protection Agency (EPA) has permitted a maximum limit of 2 ppb Hg^2+^ in potable water.^90^ According to the WHO data, the mercury level in the ground and surface water is about ∼5.00 × 10^−7^ M (μg L^−1^), and in the case of daily intake, in food it is in the range of 2 to 20 μg per day.^91^ Moreover, this metal ion generally shows fluorescence quenching due to its paramagnetic nature and strong spin–orbit coupling.^92^

Consequently, the design and synthesis of probes for Hg^2+^ have increased in importance in recent times. The development of new chemosensors for d-block metal ions or transition metals, where heavy metal ions such as Hg^2+^ are found, is of great interest given that these species play a crucial role in different environmental areas and biological systems.^93^ This section of this review focuses on the design, development, and evaluation of probes based on N-heterocycles for sensing Hg^2+^, which is one of the most toxic cations considering environmental pollution.^94^ In general, the probes analyzed below show structural variations to favor the sensing process *via* the chelation of Hg^2+^. In this case, the introduction of oxygen and sulfur atoms generates specific changes in sensitivity and selectivity in an appreciable manner. Similarly, as in Section 2, probes having 5- and 6-membered N-heterocycles are divided in this section. In these chemosensors, the N-heterocycle acts as a recognition site, in some it acts as a linker, and others it has fluorophore functions.

### 3.1. Pyrrole derivates

Although there are many probes to detect Hg^2+^ based on pyrrole derivatives, the examples using the monocyclic ring are very scarce in recent studies. Consequently, the studied pyrroles in this contribution have fused structures such as indoles, carbazoles, and BODIPYs. These pyrrole derivatives are characterized by their exceptional synthetic and functional versatility, high quantum fluorescence yields, and the low limits of detection they can achieve.^95–103^

#### 3.1.1. Indole derivates

**Fig. 22:**
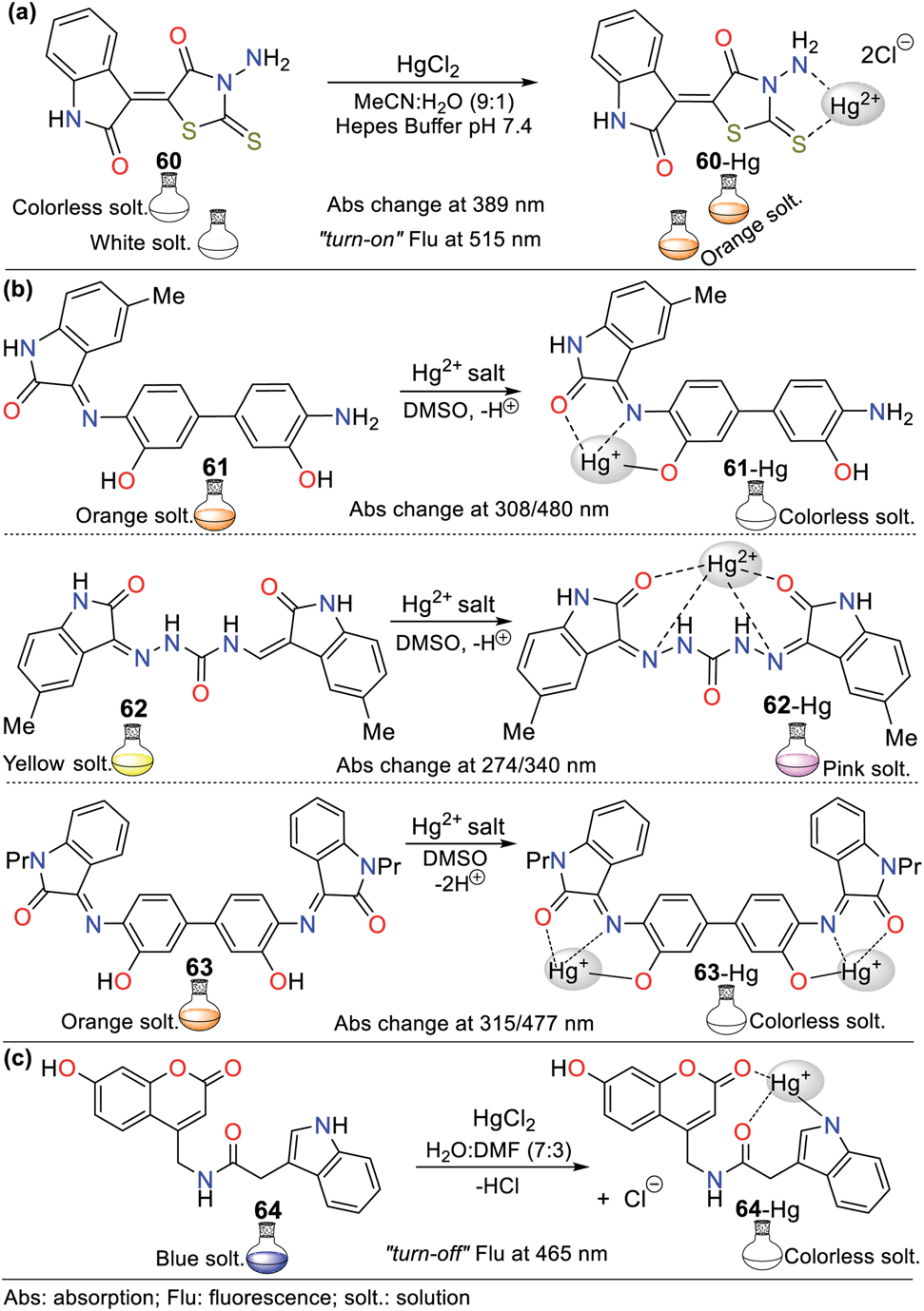
Indole-based chemosensors (a) 60, (b) 61/62/63, and (c) 64.

The combination of pyrrole with benzene rings can strengthen its photophysical properties. Accordingly, Bayindir developed the colorimetric and fluorometric probe 60 bearing a rhodanine core and possessing properties to sense Hg^2+^ (Fig. 22a).^95^ In this study, solutions of 60 (10^−2^ M) and Hg^2+^ (HgCl_2_ 10^−2^ M) were prepared in CH_3_CN : H_2_O (9 : 1) with HEPES buffer solution (pH 7.4). This probe exhibited an absorption spectrum with the typical oxindole and rhodanine absorption bands, including a strong band at 272 nm and a weak band at 390 nm. Upon the addition of Hg^2+^ to 60, the band at 272 nm increased to 275 nm with a blue/redshift and that at 390 nm redshifted to 415 nm with a substantial increase in absorbance. A significant color change from colorless to orange was observed with the naked eye. Likewise, 60 showed weak fluorescence at 515 nm (*λ*_exc_ = 390 nm), and after the successive addition of Hg^2+^, this emission band was enhanced. Furthermore, studies related to the Job's plot showed that the stoichiometry of complex 60-Hg is 1 : 1 with a binding constant of 2.15 × 10^4^ M^−1^. The LOD value of 60 was also calculated to be 3.36 × 10^−6^ M for the Hg^2+^ recognition.

Similarly, Trivedi *et al.*^96^ synthesized and characterized probes 61, 62, and 63 having an isatin core as the chromophoric unit and a 3,3′-dihydroxybenzidine moiety, possessing hydroxy, imine, and amide groups as binding units for the detection of both Hg^2+^ and AsO_2_^−^ (Fig. 22b). Chemosensor 61 showed a color change from orange to colorless for Hg^2+^ and orange to aqua-blue for AsO^2−^, 62 changed from yellow to pink for Hg^2+^ and 63 revealed selectivity towards Hg^2+^, changing from orange to colorless and orange to aqua blue for AsO^2−^. All experiments were carried out in DMSO, where the chemosensors displayed negligible absorption changes for 60 min.

Probes 61 (8.00 × 10^−5^ M), 62 (5.00 × 10^−5^ M), and 63 (4.00 × 10^−5^ M) showed two absorption bands at 308/480 nm, 274/340 nm, and 315/477 nm, respectively, which match the π–π* and n–π* transitions. The stoichiometry, LODs, and binding constants for the probe–Hg^2+^ complexes were determined. The analysis of the probes indicated a 1 : 1 (61 and 62) and 1 : 2 (63) binding stoichiometry; and LODs of 4.13 × 10^−6^ M, 4.65 × 10^−6^ M, and 3.93 × 10^−6^ M for; and binding constants of 2.51 × 10^3^ M^−1^, 1.34 × 10^4^ M^−1^ and 4.94 × 10^9^ M^−2^, respectively, for 61 to 63. DFT calculations were carried out, which showed that the charge transfer occurs from the molecule to mercury atom in 61-Hg, that is, ligand–metal charge transfer (LMCT) was observed, whereas the other sensor molecules, 62 and 63, exhibited MLCT.^96^

Similar to the last example, Joshi *et al.*^97^ synthesized the fluorescent probe 64 (Fig. 22c). UV-vis/fluorescence studies were carried in DMF : H_2_O (3 : 7) with different metal ions (5 equiv.) and a probe concentration of 10^−4^ M. This probe exhibited a strong absorption band at 294 nm with a weak hump at 344 nm and an emission band at 465 nm (*φ* = 22%, *λ*_exc_ = 340 nm). In addition, the fluorescence intensity change *versus* pH value was evaluated, showing that the band at 465 nm remained unperturbed in the pH range of 4.5 to 7.5, but its intensity increased at a higher pH value. These results indicate that the pH range of 4.5 to 7.5 is suitable for exploring sensing ability in ambient conditions. Upon the addition of Hg^2+^ (5 equiv.), a redshift of 3 to 4 nm occurred in the absorption band at 294 nm, but no change was observed with the addition of other metals; similarly, fluorescence quenching (*φ* = 5) due to ICT phenomena occurred *via* the complexation of 64 and Hg^2+^. This fluorescence quenching could be due to the excitation energy transfer from the probe to the metal d-orbital and/or metal to probe charge transfer.

For probe 64, a decrease in fluorescence intensity was observed at pH 4 because the N-indolic protonation generates a hindrance for binding to Hg^2+^ ions. The fluorescence intensity of 64 at higher pH increased with Hg^2+^ in alkaline solution. The authors explained that it also showed quenching, which could be due to decomplexation, and later the formation of mercury hydroxide. Based on the fluorescence titration curve using 3*σ*/*m*, the LOD was calculated to be 1.43 × 10^−7^ M in the Hg^2+^ concentration range of 0 to 3.00 × 10^−5^ M. In addition, the linearity of the Benesi–Hildebrand plot indicates a 1 : 1 stoichiometry between 64 and Hg^2+^, as confirmed by the ESI-MS analysis of the complex 64-Hg, which presented an association constant of 6.4 × 10^3^ M^−1^. Using ^1^H NMR studies and DFT calculations, the complexation mechanism was proposed, which could be the probable balance between the NH-amidic and NH-indolic donor centers given that Hg^2+^ shows greater affinity.^97^

#### 3.1.2. Carbazole derivates

**Fig. 23:**
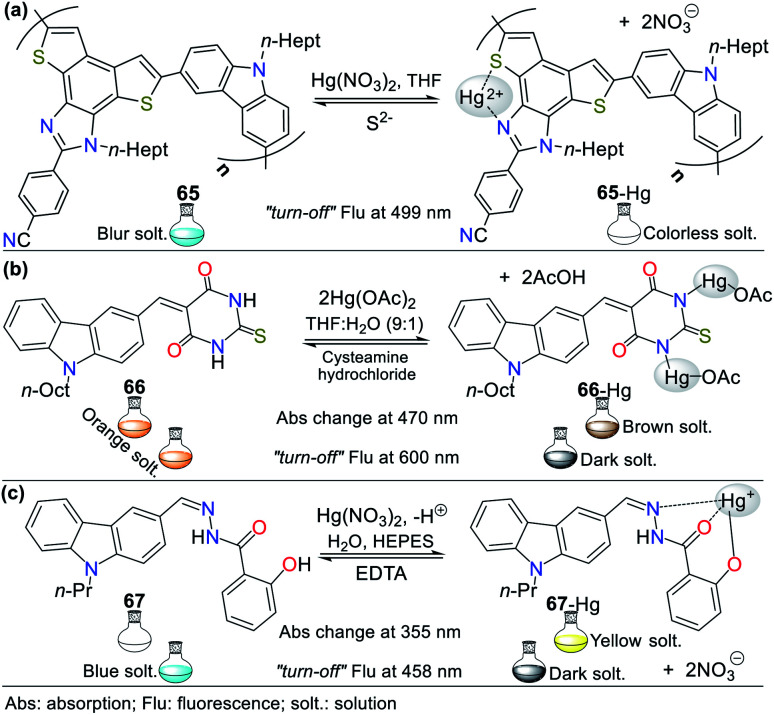
Carbazole-based probes for Hg^2+^ sensing (a) 65, (b) 66, and (c) 67.

Following the same design guide, fluorochromophores with fused systems such as carbazole endow probes better photophysical behavior. For example, Giri *et al.*^101^ obtained poly(benzodithienoimidazole-carbazole) copolymer 65*via* Pd(0)-catalyzed Suzuki coupling polymerization (Fig. 23a). Polymer 65 exhibited absorption maxima at 305 and 361 nm in (THF) solution, and strong cyan to blue emission centered at 499 nm (*λ*_exc_ = 361 nm) with quantum yields (*φ*) in the range of 22% to 21% in THF. Similarly, in the photophysical studies, a redshift of 5 nm (in the absorption spectrum) and 30 to 40 nm (in the emission spectrum) were observed due to the enlarged π-conjugation of the polymer backbone in the solid-state.

By adding alkaline-earth and heavy/transition metal ions to 65, no major changes its absorption intensity were noticed, but with the treatment of 1 equiv. of Hg^2+^, a notable fluorescence “*turn-off*” response was observed; furthermore, 65 exhibited partial quenching upon the addition of Cu^2+^. In addition, excellent selectivity was observed towards different Hg^2+^ salts irrespective of the counterions and in the vicinity of other competing metal ions. The fluorescence intensity of 65 with Hg^2+^ remained unperturbed in the pH range of 5.4 to 8.5, establishing its capacity to be used under physiological conditions. The stoichiometric ratio of 65-Hg was 1 : 1, as determined by the Job's plot and confirmed by MALDI-TOF, with a binding constant of 4.75 × 10^5^ M^−1^ and LOD of 1.35 × 10^−7^ M. The plausible sensing process could be *via* chelating quenched fluorescence (CHQF), with reversibility by adding S^2−^.^101^

Furthermore, in 2018, Kala *et al.*^100^ developed the selective “*turn-off*” probe 66 for sensing Hg^2+^ in acetonitrile and THF : H_2_O (9 : 1) based on a carbazole–thiobarbituric acid conjugate system (Fig. 23b). This probe has characteristics of a π-conjugated donor–acceptor system with an ICT absorption band. In addition, 66 has a carbazole-based locally-excited (LE) state and an ICT state due to its pre-twisted form, having a twisted excited state giving rise to a carbazole-type emission at 425 and 600 nm, respectively. In acetonitrile, the orange solution turned dark brown with a redshift due to the change in the electron affinity of the pyrimidine moiety on complexation with Hg^2+^ ions.

Moreover, the orange-colored solution of 66 changed to light brown, which could be observed by the naked eye, and from orange to dark fluorescence in a 9 : 1 THF : H_2_O mixture. The results showed that the LOD was 13.35 × 10^−9^ M for fluorescence in MeCN (*λ*_exc_ = 404 nm) and 53.34 × 10^−9^ M in 9 : 1 THF : H_2_O solution (*λ*_exc_ = 360 nm), the Job's plot showed a 1 : 2 binding stoichiometry, and binding constant was 1.49 × 10^9^ M^−2^ by absorption spectroscopy and 0.58 × 10^9^ M^−2^ by fluorescence spectroscopy, which indicate that 66 is very sensitive to Hg^2+^ ions. Moreover, the fluorescence was restored by adding cysteamine hydrochloride, indicating that 66 can serve as a reusable probe.^100^

Finally, Yin *et al.*^99^ reported the hydrazide–carbazole system 67, which showed AIE enhancement behavior for the selective sensing of Hg^2+^ and Al^3+^ in aqueous medium. This colorimetric/fluorimetric probe showed a weak fluorescence band at 438 nm in DMSO (10^−5^ M, *φ* = 0.84%), while in DMSO : H_2_O (1 : 99, v/v, HEPES buffer, pH 7.4), it emitted intense blue light under a UV lamp (365 nm) with a redshift from 438 to 458 nm (*φ* = 3.03%). Similarly, 67 (10^−6^ M) in ∼100% aqueous solution (HEPES buffer, pH 7.4) showed two absorption bands at 355 and 310 nm, which are related to π–π* and n–π* transitions, respectively. The color change from colorless to yellow was only observed in the presence of Hg^2+^ at 355 nm, indicating that 67 coordinates with Hg^2+^. In addition, the presence of 2 equiv. of Hg^2+^ led to complete fluorescence quenching at 458 nm, which could be related to the chelation-enhanced fluorescence quenching (CHEQ) effect (Fig. 23c). In contrast, upon the addition of Al^3+^ (2 equiv.) to 67, a remarkable “naked-eye” fluorescence color change from blue to green and emission enhancement with a redshift in emission (18 nm) were observed. This result is associated with C=N isomerization and inhibition of the PET process.

The binding stoichiometry for 67-Hg^2+^ and 67-Al was 1 : 1 for both, as determined by Job's method, with association constants of 4.06 × 10^4^ M^−1^ and 2.92 × 10^4^ M^−1^, respectively. The LODs were calculated to be 1.47 × 10^−8^ M for Hg^2+^ and 4.72 × 10^−8^ M for Al^3+^, which are much lower than the WHO guidelines for drinking water. Probe 67 (10^−5^ M) showed excellent stability within a wide pH range (2 to 11), as well as its complexes (pH 3 to 10), which makes 67 suitable for application in biological systems, with good sensibility (15 s). The sensing process was sustained several times with the alternating addition of Al^3+^ and F^−^, and similarly after the addition of Hg^2+^ and EDTA systems, demonstrating the reversibility and regeneration of 67. Lastly, 67 was successfully applied in different samples such as soil, environmental water, food sample analysis, and live-cell imaging, demonstrating its great potential in environmental and biological applications.^99^

#### 3.1.3. BODIPY derivatives

There are many types of fluorophores for the detection of metals, and in various reported works, some N-heterocyclic cores with excellent photophysical properties are highlighted. In this respect, BODIPY derivative dyes possess favorable characteristics, such as intense absorption and fluorescence in the visible spectral region, high molar absorption coefficients, and high quantum yields.^98^

**Fig. 24:**
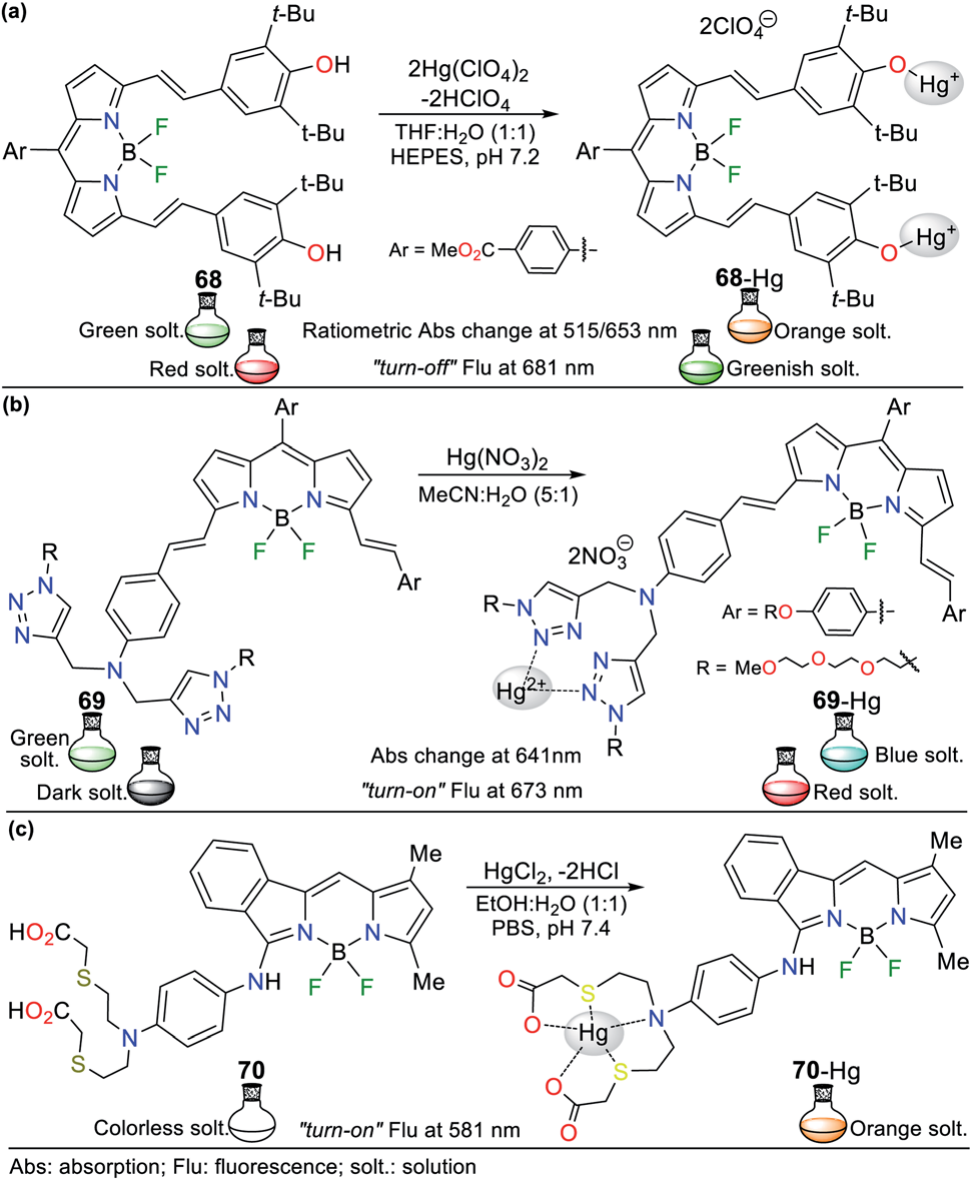
Probes having a BODIPY fluorophore (a) 68, (b) 69, and (c) 70.

In 2019, Xue *et al.*^102^ reported that probe 68 was highly selective and sensitive for recognizing Hg^2+^, which is based on 4-hydroxystyryl-substituted BODIPYs (Fig. 24a). The photophysical properties of 68 were measured in THF : H_2_O (1 : 1; HEPES 10^−4^ M, pH 7.2), exhibiting an intense absorption band at 653 nm with a molar absorption coefficient (*ε*) of 1.40 × 10^5^ M^−1^ cm^−1^ due to the low energy S_0_ → S_1_ transition of the BODIPY core and two weaker bands at 322 nm and 377 nm with a molar absorption coefficient (*ε*) of 3.30 × 10^4^ M^−1^ cm^−1^ and 7.50 × 10^4^ M^−1^ cm^−1^, respectively. This probe showed an emission band at 681 nm with a quantum yield of 89%, indicating the high fluorescence of the dye (*λ*_exc_ = 650 nm).

With the addition of Hg^2+^ to a solution of 68, a redshift in its bands was observed with a color change in the solution from light green to orange. However, the addition of different metal ions to a solution of 68 did not show noticeable changes due to the low affinity of these ions, except Hg^2+^. When Hg^2+^ ions were added, the two intense peaks located at 433 nm and 801 nm were replaced by a new intense peak located at 515 nm, which was similar to absorption spectrum of BODIPY, indicating that the Hg^2+^ ions broke the ICT process from the styryl groups to the BODIPY core. Probe 68 in THF : H_2_O (1 : 1; HEPES 10^−4^, pH = 7.2) showed bright red fluorescence with an intense emission band around the NIR region at 681 nm. Upon the addition of different ions, the emission intensity of the band at 681 nm remained the same, except in the presence of Hg^2+^. In this case, the fluorescence emission was almost quenched, with a change in color from red to green, which could be observed by the naked eye. The LOD of 68 was estimated to be 7.00 × 10^−8^ M, and the association constant was 4.30 × 10^3^ M^−1^. Finally, the Job's plot indicates that Hg^2+^ is coordinated to 68 in a 2 : 1 ratio.^102^

Similarly, Huang *et al.*^103^ developed the NIR styryl-BODIPY probe 69 for the detection of Hg^2+^ and Cu^2+^ (Fig. 24b) in CH_3_CN : H_2_O (5 : 1). This probe showed a strong red-shift for the band at 663 nm due to the ICT process and the other peaks at 614 and 385 nm. In addition, an NIR fluorescence emission was observed at 730 nm (*λ*_exc_ = 620 nm), which was largely blue-shifted from 730 to 672 nm upon the addition of Hg^2+^, with a remarkable fluorescence enhancement by the naked eye, corresponding to the inhibition of ICT. The binding mode of 69-Hg was 1 : 2, as determined by the Job's plot of the fluorescence at 673 nm, with a binding constant of 1.0 × 10^9^ M^−2^ and LOD of 9.00 × 10^−8^ M. An advantage of this probe was that with its introduction in HeLa cells for living cell imaging, it could discriminate Hg^2+^ and Cu^2+^ ions *via* two NIR fluorescence emission channels.

Compound 70 obtained by Zhou and co-workers^104^ is very similar to the previous examples. These authors developed the fluorescent probe “*turn-on*” 70, having a benzo-BODIPY fluorophore with a carboxyl-thiol metal bonding receptor for Hg^2+^ (Fig. 24c). This probe showed a visible absorption band at 512 nm and was almost nonfluorescent at 581 nm (*φ* = 0.2%) due to the efficient PET process quenching of the excited benzo-BODIPY fluorophore by the electron-donating nitrogen atom from the carboxyl-thiol metal bonding receptor. After the addition of Hg^2+^, the emission band rapidly increased with a marked color change from dark to orange due to the PET-OFF pathway between BODIPY and carboxyl-thiol (*φ* = 12% with 100 equiv. of Hg^2+^). By titration with Hg^2+^, the binding constant was determined to be 1.16 × 10^4^ M^−1^ with a 1 : 1 stoichiometry for 70-Hg in the pH range of 4.5 to 9.1, indicating that 70 can be used in neutral natural systems. The LOD was calculated to be 5.70 × 10^−9^ M. The results of this study may be considered in the future to use 70 for the detection of Hg^2+^ in real samples and other biological uses.

### 3.2. Imidazole derivatives

A large number of imidazole derivatives has been developed for different uses in medicine. Imidazole derivatives have occupied a significant place in photophysical investigations in recent years.^1–4,69,70^ In this section, the aim is to present some of the works reported on the synthesis of probes for the recognition of Hg^2+^, bearing an imidazole core as a recognition site.^105–108^ For instance, Gao *et al.*^105^ obtained Schiff-base 71 to detect Cu^2+^ (“*turn-off*”) and Hg^2+^ (“*turn-on*”), bearing benzimidazole and coumarin fluorophores. This probe showed a weak fluorescent emission at 428 nm (*φ* = 0.9%) due to the possible C=N isomerization-rotation deactivation process and high selectivity for Hg^2+^ with a light-blue emission (*φ* = 15%). An increase in fluorescence was observed with a redshift of 20 nm to 448 nm upon the addition of 5 equiv. of Hg^2+^, monitoring for 30 min in 2.00 × 10^−4^ M HEPES buffer/DMSO solution (9 : 1, pH 7.2).

**Fig. 25:**
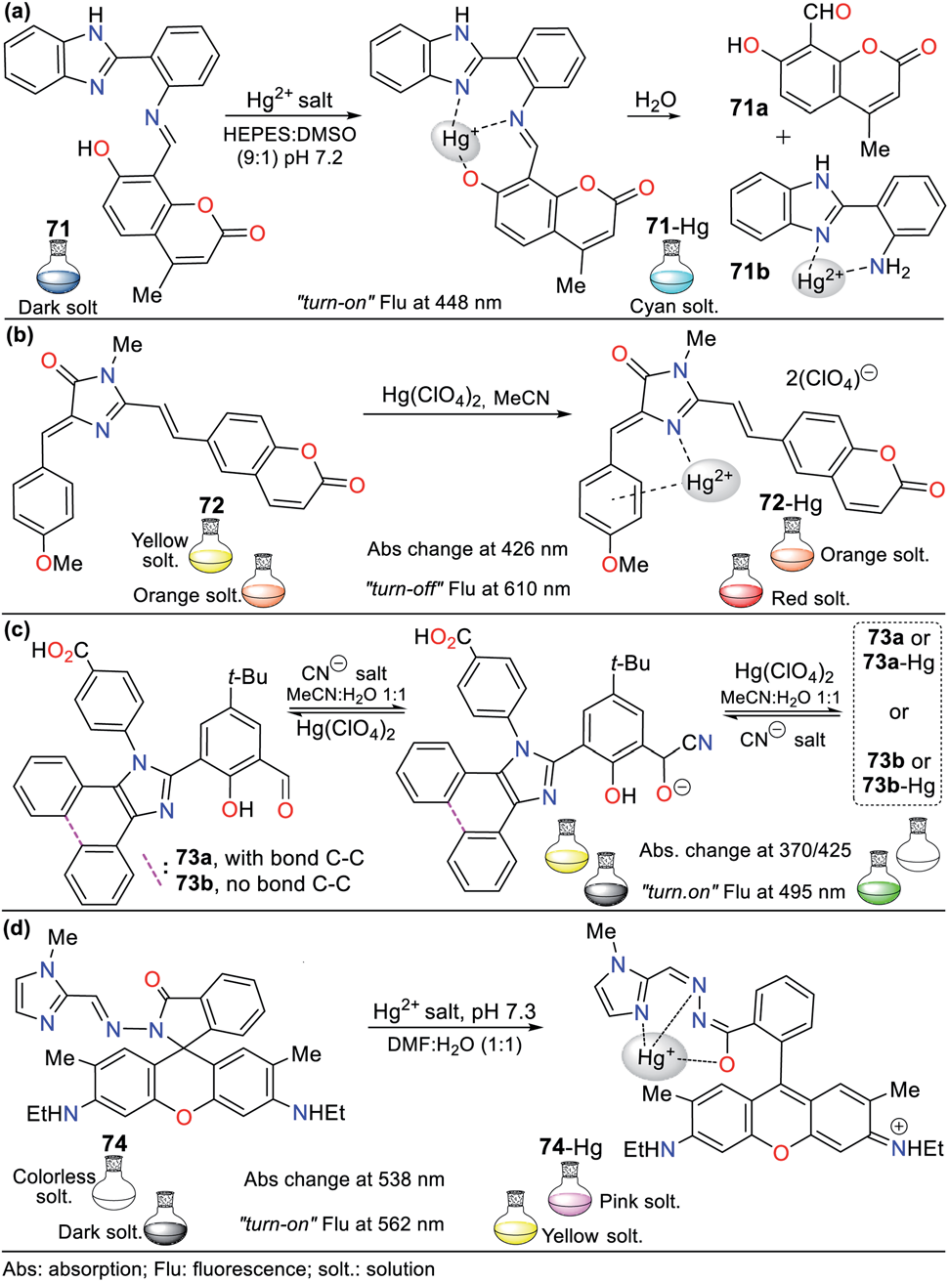
Chemosensors having imidazole (a) 71, (b) 72, (c) 73, and (d) 74.

The linear relationship of the fluorescence titration studies and Job's plot suggests a 1 : 1 binding for 71-Hg with an association constant of 8.75 × 10^4^ M^−1^ and LOD of 7.00 × 10^−8^ M. The fluorescence of 71 remained stable in the pH range of 6 to 12, which was greatly enhanced in the pH range of 6 to 8 with a maximum at pH 7, showing its promising application in biological systems. By ^1^H NMR and FTIR spectral analysis, the possible mechanism between Hg^2+^ and 71 was proposed (Fig. 25a), where Hg^2+^ may be first coordinated with 71. Then, the imine bond of the complex may be more easily attacked by a water molecule to produce compounds 71a and 71b. This probe was used in the imaging of U87 MG human primary glioblastoma cells, indicating its promising application in living cells.^105^

Motivated by the advantage of fluorescent protein derivatives for detecting pH, RNA, and human serum albumin, Ramanathan and Singh obtained 72, an analog of the red fluorescent protein (RFP) (Fig. 25b), which was recognized as a probe for Cu^2+^ and Hg^2+^.^106^ This probe has a fluorescent coumarin moiety to enhance the emission property and showed an absorption band at 426 nm (*ε* = 48.406 M^−1^ cm^−1^) and an emission band at 550 nm. Upon the addition of Hg^2+^ (10 equiv.) to 72 in acetonitrile (10^−4^ M), a color change from yellow to orange was perceived by the naked eye. Similarly, the emission band suffered a redshift from 550 (*φ* = 6.7%) to 610 (*φ* = 6.3%) nm with fluorescence “*turn-off*” due to the enhanced spin–orbit quenching, and a color change from bright orange to red. The formation of complex 72-Hg took place in a 1 : 1 ratio with a binding constant of 3.8 × 10^5^ M^−1^ and LOD_Flu_ of 2.00 × 10^−11^ M. A static quenching pathway was observed given that the decay time was 7.6 ± 0.3 ns both in the presence and absence of Cu^2+^ and Hg^2+^ ions, showing that the formation of a ground state non-emissive complex occurs with both ions. Similarly, the coumarin moiety did not participate in the binding with the metal center but significantly favors the LOD of 72 as a colorimetric chemosensor.

In 2018, Emandi *et al.* described the synthesis of two fluorescent probes for the reversible detection of Hg^2+^ and CN^−^ in MeCN : H_2_O (1 : 1, 10^−5^ M).^107^ These dual probes are fused imidazole 73a and tetraarylimidazole 73b, which displayed absorption bands at 370 and 345 nm with strong fluorescence at 495 nm (*φ* = 19%) and 491 nm (*φ* = 12%), respectively. The recognition studies indicated the formation of cyanohydrins 73a-CN (LOD = 8.0 × 10^−7^ M) and 73b-CN (LOD = 1.2 × 10^−6^ M) upon the addition of CN^−^ to the probe solution. Later, upon the addition of Hg^2+^, a reduction in the absorption band at 425 nm was observed with an increase in the band at 370 nm; equally, the fluorescence intensity markedly increased at 495 nm, turning the solution green. These results suggest the binding of Hg^2+^ to 73a-CN, which was confirmed by ^1^H NMR and supported by ESI-MS studies. Similar optical changes were observed for 73b. CN^−^ induces notable fluorescence changes by “*turn-off*” behavior, while the subsequent addition Hg^2+^ results in fluorescence “*turn-on*”, suggesting a reversible and reusable probe for around ten cycles. Thus, the binding stoichiometry was probably a 1 : 1 ratio (Fig. 25c).

Additionally, Rao *et al.*^108^ developed chemosensor 74, having an *N*-methylimidazole linked by a hydrazide linker to a rhodamine 6G fragment (Fig. 25d). This probe presented exceptional sensing ability in an acid or neutral medium, working by three chelation points, as confirmed by the ^1^H NMR and ESI-MS spectra (*i.e.*, –O, =N, and =N). The absorption spectra of 74 in DMF : H_2_O (1 : 1, pH 7.3) showed two peaks at 267 and 309 nm, while with an increase in the concentration of Hg^2+^, another band emerged at 538 nm due to the ring-opening spirolactam; equally, the emission spectra offered a new intense band at 562 nm. Upon the addition of Hg^2+^, the color of 74 changed from colorless to pink rapidly in daylight with yellow fluorescence “*turn-on*”. The binding constant was 5.12 × 10^5^ M, indicating a strong interaction in 74-Hg with a binding stoichiometry of 1 : 1 by the Job's plot. The practical use of this molecular probe as electrospun nanofiber test strips to recognize Hg^2+^ in aqueous media was accomplished.

### 3.3. Pyrazole derivatives

In recent years, pyrazole derivatives have attracted significant attention in organic and medicinal chemistry because of their usefulness in preparing bioactive compounds or ligands for the formation of coordination complexes with different metal centers. There are some examples of pyrazole-based probes for the detection of Hg^2+^, in which the ring acts as a signaling unit, being part of a fused system; however, in other cases, pyrazoles and pyrazolines also act as recognition sites. Remarkably, in all the examples presented in this section, the chemosensors have blue fluorescence, which is quenched when they bind to Hg^2+^ ions.^109–113^

**Fig. 26:**
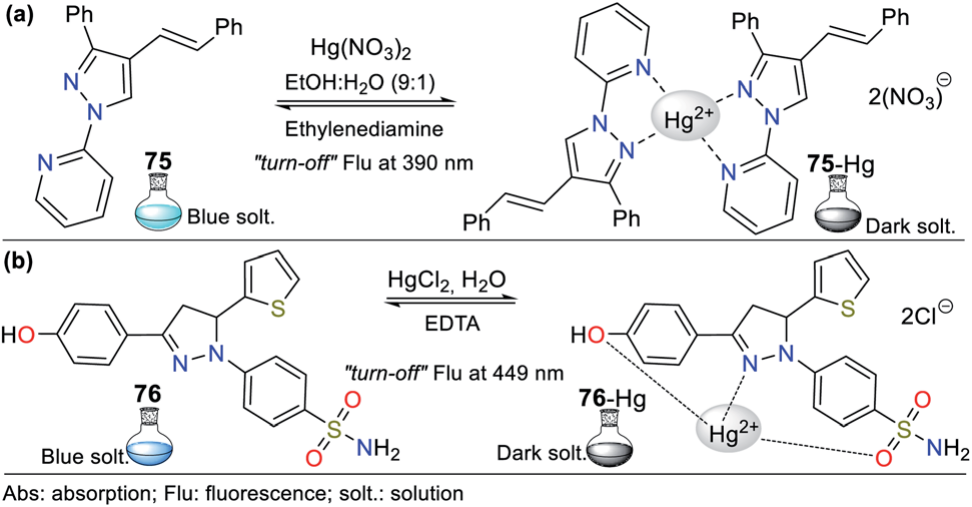
Chemosensors for sensing Hg^2+^ having pyrazol (a) 75 and (b) 76.

Taking advantage of our knowledge on pyrazole derivatives^2,13,20^ and considering their important electronic properties, our group synthesized a family of 4-styrylpyrazoles and applied 3-phenyl-1-(2-pyridyl)-4-styrylpyrazole (75) as a chemosensor to detect Hg^2+^.^109^ The 2-pyridyl acceptor group in 4-styrylpyrazoles favors the D–π–A system and their dipole moment, generating fluorophores with strong blue-light emission (quantum yields of up to 66%). Consequently, probe 75 (4.0 × 10^−6^ M in EtOH : H_2_O) was selected to evaluate its metal ion sensing capacity, where only the presence of Hg^2+^ induced significant fluorescence quenching. In the emission studies, with an increase in the concentration of Hg^2+^, the fluorescence was completely turned off with the addition of 200 equiv. of Hg^2+^ to the solution. The binding mode of 75-Hg was 2 : 1, as determined by the Job's plot of the fluorescence at 390 nm, with an LOD of 3.10 × 10^−7^ M (*R*^2^ = 0.9950) and an LMCT process (Fig. 26a).

Pyrazolines can also be applied for sensing Hg^2+^, for example, Bozkurt and Gul reported the development of probe 76.^110^ This chemosensor was obtained with diverse substituents in the molecule such as 4-hydroxyphenyl at position 3, thiophene-2-yl at position 5, and benzenesulfonamide at position 1 (Fig. 26b). In addition, 76 showed an absorption band at 355 nm and a relatively high-intensity emission at 449 nm (*φ* = 68% in water), which remained unchanged upon the addition of various metals to the solution. However, both the quantum yield (*φ* = 16%) and the fluorescence lifetime of 76 decreased in the presence of Hg^2+^ (from 0 to 10^−4^ M), indicating that this analyte quenched the fluorescence. The results from the Job's plot indicated the interaction ratio of 76-Hg was 1 : 1, the calculated LOD was 1.45 × 10^−5^ M in the linear range of (2 to 20) × 10^−5^, and the binding constant was 8.06 × 10^4^ M^−1^. The EDTA test confirmed the reversibility of this probe, showing that detecting Hg^2+^ is reversible several times. This probe was applied for the analysis of real water samples, providing excellent LOD results (4.70 × 10^−7^ M).

**Fig. 27:**
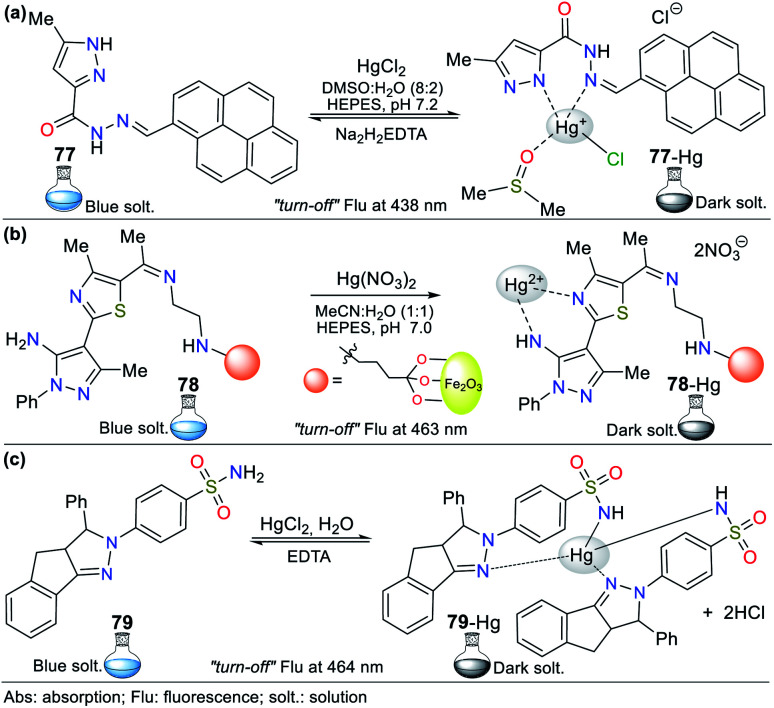
Probes for Hg^2+^ sensing having pyrazole (a) 77, (b) 78, and (c) 79.

Naskar *et al.*^111^ were inspired by the photophysical benefits of pyrene derivatives to obtain pyrene–pyrazole probe 77 for sensing Hg^2+^ (Fig. 27a). The successive addition of Hg^2+^ to the probe solution from 0 to 5 × 10^−5^ M in DMSO : H_2_O (8 : 2) with HEPES buffer at pH 7.2 caused a gradual decrease in the absorption intensity and confirmed the existence of a new product due to the interaction of 77-Hg. Probe 77 showed intense green fluorescence when excited at 374 nm (*φ* = 21%). Typical emission bands at 418 and 438 nm were observed, which are related to the monomer and excimer emissions of the pyrene moiety, respectively. The addition of Hg^2+^ quenched the emission (*φ* = 2%), and fluorescence was tested as a function of pH, showing that in the pH range of 4 to 8, the solution of 77 exhibits negligible fluorescence in the presence of Hg^2+^, which is suitable for biological applications.

According to the Job's plot studies, it was determined that Hg^2+^ forms a 1 : 1 complex with 77 with a binding constant of 7.10 × 10^5^ M^−1^, as confirmed by NMR experiments, ESI-MS spectrometry, and DFT calculations. This probe presented reversibility upon the alternating addition of Hg^2+^/Na_2_H_2_EDTA, making 77 a reversible Hg^2+^ sensor with an LOD of 9.20 × 10^−9^ M at 438 nm. The DFT calculations revealed that three rotamers are accessible to bind to Hg^2+^ ions by rotating the pyrazole ring and carbonyl groups, which validated the important role of the pyrazole ring in 77. By testing the utility of 77 in live-cell imaging, the studies established that the 77 could detect cellular cytoplasmic Hg^2+^ in HepG2 cells.^111^

Several reported nanocomposites can be used for the removal of heavy metals in contaminated waters. In this case, Mir *et al.*^112^ studied the detection of Hg^2+^ using fluorescent probe 78 bearing a pyrazole derivative functionalized with Fe_3_O_4_@SiO_2_. Probe 78 has the advantage of easy separation by an external magnetic field because of its magnetic core (Fig. 27b). The solution of 78 (10^−5^ M) in CH_3_CN : H_2_O (1 : 1) with HEPES buffer (10^−4^ M, pH 7) showed an emission band at 463 nm (*λ*_exc_ = 225 nm) and no change upon the addition of toxic metals except for a slight change in intensity for some cations. However, a notable variation for Hg^2+^ was observed with fluorescence quenching at 463 nm. The decrease in fluorescence intensity could be attributed to well–known processes such as enhanced spin–orbit coupling or energy/electron transfer of Hg^2+^. The coordination geometry, nitrogen affinity, and ion size and charge are vital for selectivity high Hg^2+^. A good linear correlation between the Hg^2+^ concentration and emission intensity in the range of 10 to 50 equiv. was observed with an LOD of 7.60 × 10^−9^ M. Computational calculations revealed nonparticipation of the pyrazole core in the coordination complex, acting as a fluorophore.

Finally, considering the advantages of pyrazolines as widely known fluorophores, Bozkurt and Gul synthesized fluorescent derivative 79 for the fluorometric detection of Hg^2+^ (Fig. 27c).^113^ Probe 79 showed two absorption bands at 278/363 nm and a relatively intense emission band at 464 nm in water. Notably, its absorption spectrum did not change in the presence of metal ions, while its fluorescence intensity decreased only with Hg^2+^ (*φ* = 5%). The LOD of Hg^2+^ was determined to be 1.60 × 10^−7^ M by a linear relationship, and the interaction ratio 79-Hg was found to be 2 : 1 according to the Job's plot analysis with a binding constant of 4.69 × 10^5^ M^−2^. The results revealed that 79 is a reversible probe with recycling capability by the successive addition of Hg^2+^/EDTA (*turn-off*/*turn-on*). The detection of Hg^2+^ was studied at different pH values, and the results showed that 79 could be used for the analysis of neutral water samples and biological applications at physiological pH. The spectral changes observed in the FTIR and ^1^H NMR studies indicated that the decrease in the emission intensity of 79 is due to the electrostatic interactions of probe–Hg^2+^. This probe was successfully used for the measurement of real water samples.

### 3.4. Pyridine derivatives

Pyridine derivatives have long been used primarily in the pesticide and pharmaceutical industries. The pyridine ring is a structural component of some natural products such as vitamins, and coenzymes, alkaloids. Pyridines have been used in supramolecular chemistry, mainly due to their properties such as basicity, water-solubility, and hydrogen bond formation, and its nitrogen atom acts as a receptor site in chemosensors. In a few cases, free pyridines act as Hg^2+^ recognition sites; however, many reversible probes are observed in fused systems, where nitrogen is part of the complex and yields greater stability. Good LOD results have been observed in other fused pyridines, which means that some probes can be applied in real and biological samples.^114–121^

#### 3.4.1. Probes having pyridine

**Fig. 28:**
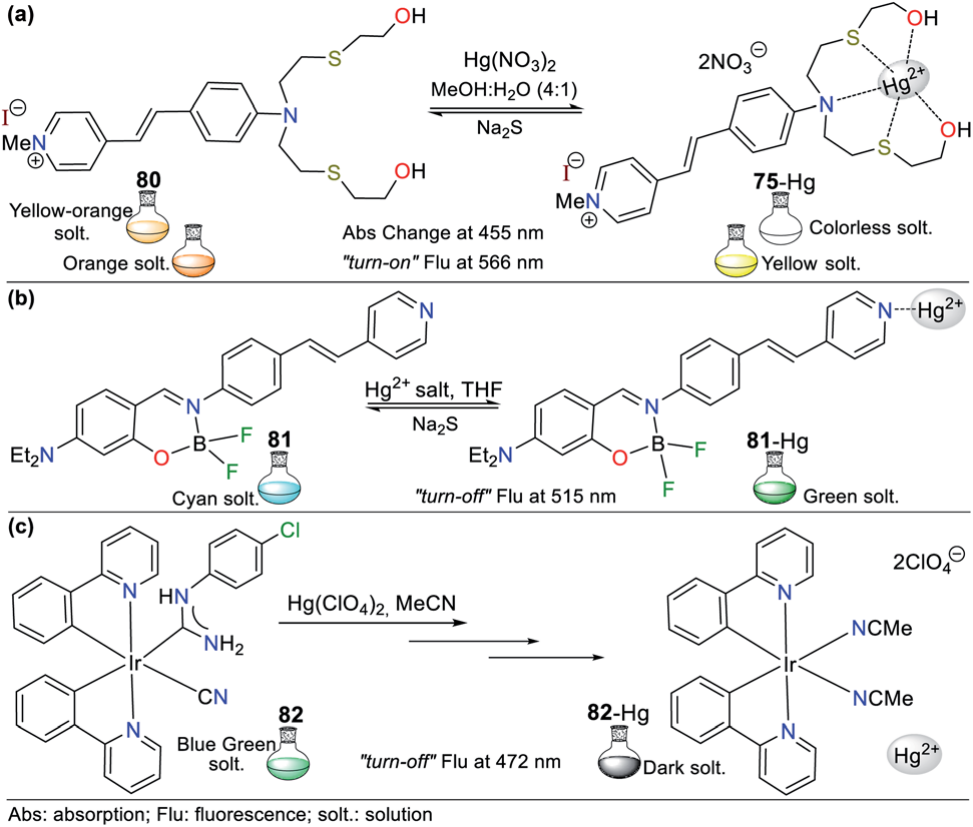
Probes for Hg^2+^ sensing having pyridine (a) 80, (b) 81, and (c) 82.

Tripathy *et al.*^114^ reported probe 80 having a D–π–A chromophore with an NS_2_O_2_ binding site (Fig. 28a). Photophysical studies of 80 (1.2 × 10^−5^ M) in water or MeOH : H_2_O (4 : 1) were carried out by adding 4 equiv. of different metal ions (4.8 × 10^−5^ M) to the solution. The solution changed from yellow-orange to colorless with Hg^2+^, but in the presence of other metal ions, it remained unaffected when observed by the naked eye. Similarly, a change from orange to strong fluorescent yellow under UV light was observed only with Hg^2+^. Compound 80 is weakly fluorescent (*φ* = 0.9%) at 590 nm, and with the addition of 4 equiv. of Hg^2+^, a notable fluorescence enhancement was observed (*φ* = 4.4%) with a blue shift in the emission band at 566 nm. This fluorescence “*turn-on*” may be explained by the principle of chelation-enhanced fluorescence (CHEF) due to the inhibition of the PET process and increased conformational rigidity. The 1 : 1 binding stoichiometry for 80-Hg was determined by the Job's method with an association constant of 1.13 × 10^5^ M^−1^ in water and 1.46 × 10^5^ M^−1^ in MeOH : H_2_O and LOD of 7.50 × 10^−6^ M. These results suggest that 80 can be applied as a reversible fluorescent probe for the selective sensing of Hg^2+^ in an aqueous medium under physiological conditions.

Alternatively, Zhang *et al.*^115^ developed probe 81, having a pyridine–organoboron complex for the detection of Hg^2+^ in THF (10^−5^ M) or gaseous acid/base upon aggregation (Fig. 28b). Probe 81 showed a strong absorption band at 415 nm due to the π–π* transition with a light-cyan fluorescence band at 480 nm, which was altered in different polar solvents together with its absorption spectrum. These results indicate that boron restricts the σ-bond rotation and isomerization process of the imine, resulting in a high fluorescence quantum yield.

Upon the addition of Hg^2+^ to a solution of 81, the emission band intensity at 480 nm was gradually reduced to generate a new band at 515 nm with a change from cyan to green, which could be observed by the naked eyes. This probe has high Hg^2+^ selectivity *via* interactions with the pyridinic N; thus, an ICT process occurs, as evidenced by the new emission band at 515 nm. The fluorescence intensity showed good linearity for Hg^2+^ in the range of (1 to 10) × 10^−5^ M with an LOD of 5.00 × 10^−8^ M. The chemical shifts in its ^1^H NMR spectrum remained unchanged with 1.0 equiv. of Hg^2+^, indicating that 81 reached saturation coordination with 1 equiv. of Hg^2+^*via* a 1 : 1 stoichiometry. When S^2−^ ions were added to the 81-Hg complex, HgS precipitated due to the stronger combining capacity of Hg^2+^ with S^2−^, confirming that the reaction mechanism is reversible and can be regenerated for three cycles (Fig. 28b).^115^

Similarly, Eremina *et al.*^116^ developed a new application *via* bis(cyclometalated) iridium(iii) species bearing an analyte. For all the complexes, their absorption spectra exhibited a strong band at 340 nm with high molar extinction coefficients (*ε* = 1.03/7.96 × 10^4^ M^−1^ cm^−1^) due to the spin-allowed π–π* ligand-centered transitions. In addition, less intense bands appeared in the region of 340–400 nm (*ε* = 0.40/9.40 × 10^3^ M^−1^ cm^−1^), which are attributed to the spin-allowed singlet MLCT transitions. These results agree with the DFT calculations for the complexes. All the complexes presented broad blue-green phosphorescence in acetonitrile after photoexcitation at different wavelengths. Complex 82 showed an 80% decrease in intensity emission at 472 nm (*λ*_exc_ = 375 nm) and weak residual luminescence upon the addition of Hg^2+^. The titration studies indicated 2 : 1 and 1 : 1 binding stoichiometries for 82-Hg according to the Job's plot with an LOD of 2.63 × 10^−7^ M. The detection mechanism was explained by the conversion of cyanide 82 to methyl isocyanide 82-Hg *via* different Hg^2+^ complexes (*i.e.*, 82′-Hg and 82′-Hg-82′), which resulted in nearly complete disappearance of its optical properties (Fig. 28c).

#### 3.4.2. Quinoline derivatives

**Fig. 29:**
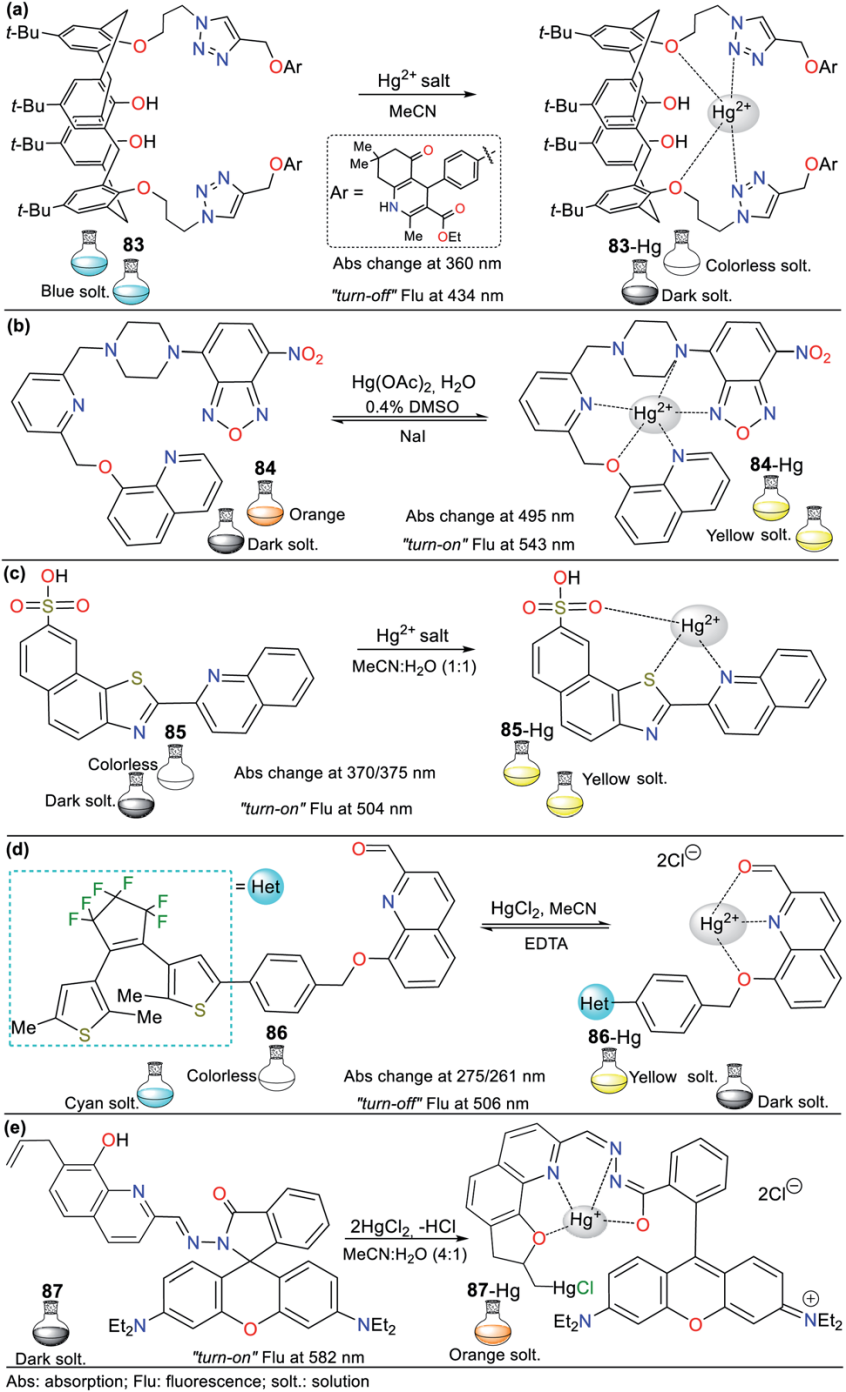
Quinoline-based probes (a) 83, (b) 84, (c) 85, (d) 86, and (e) 87.

Khan *et al.* obtained triazole derivative 83 by joining a fluorescent quinolone-appended calix[4]arene, which is highly selective for sensing Hg^2+^ (Fig. 29a).^117^ The absorption spectrum of 83 (3.00 × 10^−5^ M in MeCN) showed a band having two peaks at 278 and 360 (*λ*_max_) nm. The absorption of 83 was significantly reduced after mixing with an aqueous solution of Hg^2+^ (3.00 × 10^−5^ M), obtaining an LOD of 2.00 × 10^−6^ M. The interaction of 83 with other metal ions was not observed. This probe was also titrated at its maximum emission wavelength (*λ*_max_ = 434 nm) with an equimolar (10^−5^ M) aqueous solution of Hg^2+^, which induced fluorescence quenching with an LOD of 5.00 × 10^−7^ M, while other metal cations remained inert. In addition, the stability of the 83-Hg complex was studied over a wide pH range (2.5 to 11.8) and deprotonation of the chelating oxygen and nitrogen atoms of 83, ensuring their availability for interactions with Hg^2+^ ions, showing a decrease in absorbance intensity at in the pH range of 7 to 9.7. The stoichiometric ratio of 83-Hg was determined to be 1 : 1 by the Job's plot. Moreover, 83 was successfully applied for the detection of Hg^2+^ in human cells.

Motivated by the versatility of quinolines, Wang *et al.*^118^ developed the “*turn-on*” probe 84 for sensing Hg^2+^ in H_2_O : DMSO (∼99 : 1), bearing nitrobenzo-oxadiazole, piperazine, pyridine, and quinoline moieties (Fig. 29b). The solution of 84 is orange, and its absorption spectrum showed a strong band at 495 nm due to the ICT process from the piperazine ring to the nitro group. After the gradual addition of Hg^2+^, the ICT band at 482 showed an increase in absorption intensity with a blue shift nm due to the reduced electron-donating ability of the piperazine moiety after the coordination of Hg^2+^, which changed the solution color from orange to yellow. Similarly, the fluorescence quantum yield increased from 1.1% to 14% at 543 nm (*λ*_exc_ = 488 nm) due to the suppression of the PET process in the complex formed, given that the piperazine nitrogen donor was no longer available; thus, the solution emission was turned on from dark to yellow coloration (pH 6 to 7.5).

The complexation mechanism of 84 with Hg^2+^ was confirmed by ^1^H NMR and DFT calculations. The LOD was found to be 1.92 × 10^−8^ M, with a 1 : 1 complex according to the Job's plot analysis and the binding constant of 84-Hg was 1.15 × 10^4^ M^−1^. The reversibility of the sensing process was established and quenched by NaI, implying the decomplexation of 84-Hg with repetitions for a minimum of four cycles.^118^ The *in vivo* detection of Hg^2+^ in living zebrafish was applied to evaluate the practical use of 84.

Similarly, Zareh Jonaghani and Zali-Boeini^119^ synthesized the fluorimetric and colorimetric probe 85 (hybrid quinoline–naphthothiazole system) for sensing Hg^2+^ in MeCN : H_2_O (1 : 1, 3.00 × 10^−5^ M). This probe showed an absorption maximum at 356 nm (*ε* = 18.100 M^−1^ cm^−1^) and a fluorescence emission at 433 nm (*φ* = 32%). Upon the addition of Hg^2+^ (2 × 10^−6^ M), compound 86 showed a redshift to 504 nm and a decline in its fluorescence intensity (*φ* = 26%) with a color change from colorless to yellow, which could be observed by the naked eye. This probe showed an excellent linear relationship between the fluorescence intensity and concentration of Hg^2+^ with an LOD of 3.42 × 10^−8^ M, while the association constant was 2.00 × 10^6^ M^−1^ (pH 5 to 8). Finally, the Job's plot showed a 1 : 1 stoichiometric ratio for the 85-Hg complex (Fig. 29c).

According to the widely reported properties in other sensor designs, Guo *et al.*^120^ developed the colorimetric and fluorescent probe 86, bearing a photochromic di-3-thienylethene with 2-formylquinoline (Fig. 29d). Probe 86 showed a sharp absorption band at 261 nm (*ε* = 4.9 × 10^4^ mol^−1^ L cm^−1^ in MeCN), which is assigned to the π–π* transitions. Upon irradiation with light at 297 nm, 86 showed a photocyclization reaction. The solution color changed from colorless to purple under natural light, but from bright fluorescent cyan (*φ* = 24.5%) to dark (*φ* = 2.1%) under UV light. This probe showed strong fluorescence at 506 nm (*λ*_exc_ = 370 nm). Moreover, when its response to Hg^2+^ was examined, its absorption spectrum exhibited a redshift from 261 to 275 nm due to the LMCT process, while in fluorescence, the band at 506 nm showed quenching until 97%, and the solution changed from bright cyan to dark. The emission was restored with the addition of EDTA, confirming that the detection mechanism is reversible. The association constant of 84-Hg was 4.01 × 10^4^ L with a stoichiometry of 1 : 1, as confirmed by ESI-MS, and the LOD was 5.63 × 10^−8^ M. According to these results, an integrated logic circuit with multiple control switches was constructed.

As the last example, Jiang *et al.*^121^ obtained the hybrid rhodamine B–hydrazonoquinoline system 87 for the dual sensing of Cu^2+^ and Hg^2+^ in CH_3_CN : H_2_O (4 : 1, v/v) (Fig. 29e). The solution of 87 (5.00 × 10^−5^ M) exhibited an absorption band at around 280 nm, but a new absorption band appeared at ∼560 nm upon the addition of Cu^2+^ or Hg^2+^ ions (2.00 × 10^−4^ M). The new band increased considerably with an increase in the concentration of these metals. The isoabsorptive point at 350 nm for Cu^2+^ and 350 nm for Hg^2+^ indicated that 87 could be used as a ratiometric probe. The probe solution (10^−5^ M) showed weak emission at 572 nm (*λ*_exc_ = 355 nm) without notable changes upon the addition of solutions of different metal ions. An increase in fluorescence was observed upon the addition of 4.00 × 10^−5^ M of Cu^2+^ or Hg^2+^, and the maximum emission shifted to 582 nm; however, a change in the pale and bright orange fluorescence could be easily observed by the naked eye under a 365 nm UV lamp.

The binding constant values were 1.45 × 10^4^ M^−1^ for 87-Cu with an LOD of 1.20 × 10^−7^ M and 4.98 × 10^6^ M^−2^ for 87-Hg with an LOD 3.20 × 10^−8^ M. The fluorescence quantum yields of 87, 87-Cu, and 87-Hg are 5.4%, 82%, and 98%, respectively. The Job's plot analysis indicated a 1 : 1 binding stoichiometry for 87-Cu and 1 : 2 for 87-Hg. The emissions at 582 nm and UV-vis absorption at 560 nm attributed to metals could induce the opening of the spirocycle of the rhodamine unit. In addition, the excited-state intramolecular proton transfer (ESIPT) of the OH group in the quinoline moiety could be inhibited by both Cu^2+^ and Hg^2+^. The emission of 87-Hg^2+^ was stronger than that with the same amount of Cu^2+^, probably due to the above-mentioned ring-opening, unique regenerative cycle of the quinoline unit in 87, and the inhibition of the PET process. Studies in live Chinese hamster lung cells suggested that 87 exhibited low cytotoxicity and could be utilized in live cells *in vivo* with minor damage to monitor Cu^2+^ and Hg^2+^.^121^

#### 3.4.3. Other pyridine derivatives

**Fig. 30:**
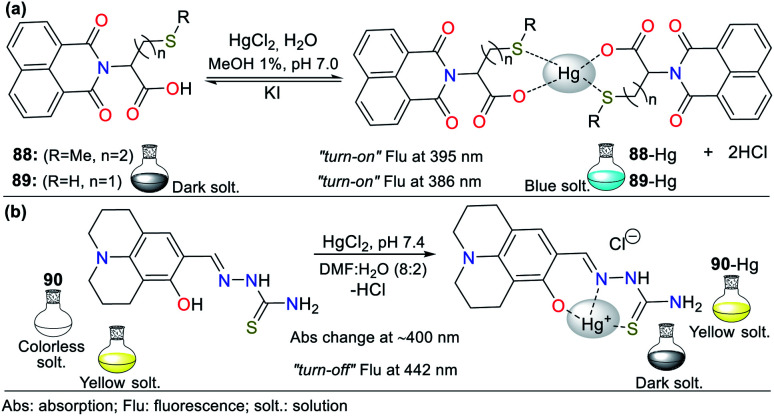
Probes bearing (a) naphthalimide 88/89 and (b) julolidine 90.

Considering the naphthalimide core and its crucial photophysical properties, Bahta and Ahmed^122^ developed, *via* a straightforward reaction, the hybrid 1,8-naphthalimide–amino systems 88 and 89 to detect Hg^2+^ (Fig. 30a). The absorption bands of 88 and 89 in methanol are located at 331 and 332 nm, while in water, they showed a redshift to 343 and 341 nm, respectively, due to the improved AIE property in aqueous medium. Similarly, the emission bands of 88 and 89 showed a redshift from 377 to 395 nm and 381 to 386 nm with an increase in the content of water, respectively. Except for Hg^2+^ analysis, the absorption spectra in MeOH : H_2_O (1 : 99) with different metal ions indicated that the intensity of the bands was unchanged. With the addition of Hg^2+^, the intensity of the bands decreased with two isosbestic points at 311/364 nm for 88 and 306/362 nm for 89, supporting the formation of complexes with this highly toxic analyte.

Conversely, the emission intensity increased, observing notable changes upon the addition of 5 equiv. of Hg^2+^ to 88 (*φ* from 8.9% to 77% at 395 nm) and 3 equiv. to 89 (*φ* from 9.5% to 75% at 386 nm) due to the CHEF effect in both probes, which was favored by the better aggregation of the complexes. In addition, the fluorescence lifetimes of 88 and 89 were 2.5 and 2.3 ns in water, while upon the addition of Hg^2+^, they changed to 7.7 and 8.5 ns, respectively, with a stoichiometry 2 : 1 for both complexes according to the Job's plot and HRMS analysis. The LODs were found to be 2.20 × 10^−8^ and 5.60 × 10^−9^ M with binding constants of 3.40 × 10^9^ and 4.60 × 10^9^ M^−2^ for 88 and 89, respectively. Notably, these probes exhibited reversibility with the addition of 10 equiv. of KI at pH 7 (HEPES-buffer solution).

Julolidine derivatives have essential photophysical properties as part of photoconductive, chemiluminescent, and chromogenic materials. Accordingly, Gupta and co-workers^123^ obtained a probe having a thiosemicarbazide moiety linked to this fluorophore (Fig. 30b), which works as a chemosensor multitarget for Hg^2+^ and Mn^2+^ in DMF : H_2_O (8 : 2). With the addition of Hg^2+^ (0 to 1.6 equiv.) to 90 (2.5 × 10^−5^ M), a redshift from 383 nm to around 400 nm was observed with a color change from colorless to yellow. These results were induced by Hg^2+^ binding to the probe, which favors LMCT from the nitrogen in the julolidine core towards the receptor site (push–pull effect).

The emission spectrum of 90 showed a band at 442 nm (*λ*_exc_ = 397 nm) without significant changes upon the addition of various metal ions (10 equiv.) to the solution (6.0 × 10^−5^ M), except for Hg^2+^, which quenched the fluorescence due to the complexing based on the MLCT-heavy metal effect. The complex stoichiometry was 1 : 1 according to the Job's plot and confirmed by ^1^H NMR titration with a formation constant of 7.73 × 10^3^ M^−1^ by the Benesi–Hildebrand method. The LOD by fluorescence was 1.50 × 10^−5^ M for Hg^2+^ and 2.00 × 10^−7^ M for Mn^2+^. This probe worked efficiently in the pH range of 5 to 10, which is within physiological limits. DFT calculations clarified the coordination and selectivity of 90 with Hg^2+^ and Mn^2+^ due to the decrease in the HOMO–LUMO energy band gap in the structures of the complexes formed. It should also be noted that 90 was used in the analysis of real water samples with satisfactory results.^123^

**Fig. 31:**
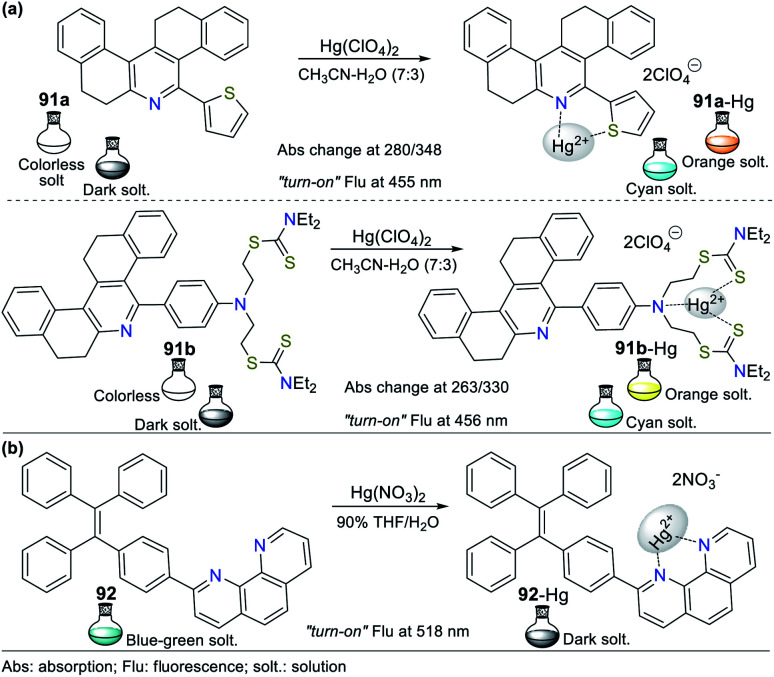
Probes for Hg^2+^ based on polycyclic systems (a) 91a/91b and (c) 92.

Alternatively, considering the particular affinity between Hg^2+^ and sulfur, Ponram *et al.*^124^ obtained the fluorescent mono-sulfur 91a and tetra-sulfur 91b probes having a tetrahydrodibenzo-phenanthridine core (Fig. 31a). The absorption spectrum of 91a showed two redshifted bands from 270/326 to 280/348 nm with the addition of Hg^2+^; similarly, 91b exhibited a redshift from 255 to 263 nm, but the band at 346 nm was blue-shifted to 330 nm. The observed changes in fluorescence colors showed that these probes detect Hg^2+^*via* “*turn-on*” for 91a and “*turn-off*” for 91b. The nature of the TICT process can explain these results given that the detection state returns to the ground state through the redshifted emission or more efficient non-radiative relaxation. Upon the addition of Hg^2+^ (1 equiv.) to 91a, the emission band at 455 nm gradually increased, while for 91b, it gradually decreased at 456 nm in MeCN : H_2_O (7 : 3). The LOD was calculated to be 9.10 × 10^−10^ M (MeCN) for 91a and 4.18 × 10^−11^ M (DMSO) for 91b with a 1 : 1 stoichiometry for both according to the Job's method. DFT calculations confirmed the sensing mechanism, showing that the LMCT phenomenon occurs between chemosensor molecule 91 and Hg^2+^.

Considering the high complexing capacity of phenanthroline with d-block metals, Tang *et al.*^125^ developed the AIE-active fluorescent probe 92, bearing a tetraphenylethene (TPE) unit, to detect Zn^2+^ (in THF) and Hg^2+^ (THF : H_2_O). The photophysical results proved the formation of nanoscopic aggregates in THF when the amount of water was increased, with a blue-green emission (*φ* from 0.8% to 80%) at 518 nm (*λ*_exc_ = 375 nm) caused by the restricted intramolecular rotation in 92-Hg (Fig. 31b). In addition, upon the addition of Hg^2+^ to the probe solution in THF : H_2_O (9 : 1), the emission intensity decreased significantly with a slight redshift and changed from bright blue-green to dark. These results were supported by ^1^H NMR studies, concluding that the coordination of 92 with Hg^2+^ was achieved using the two nitrogen atoms in the phenanthroline ring. The stoichiometry of the 92-Hg complex was 1 : 1, as confirmed by MS analysis with a quenching constant and LOD of 5.58 × 10^7^ M^−1^ and 2.55 × 10^−9^ M, respectively. Remarkably, hybrid system 92 also was used as a fluorescence “*turn-on*” probe for the detection of Zn^2+^ in THF with an LOD of 1.24 × 10^−6^ M. In addition, 92 was also studied in the solid-state, demonstrating reversible mechanochromic behavior with the fluorescent of colors blue–green–blue.

**Fig. 32:**
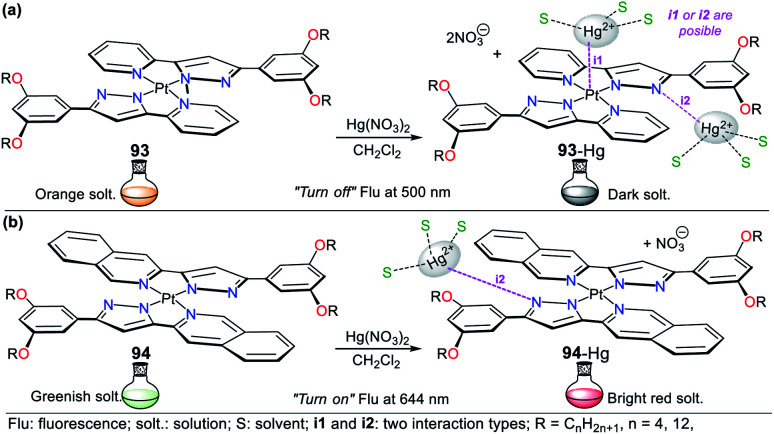
Pt(II) metallomesogens for Hg^2+^ sensing (a) 93 and (b) 94.

Ultimately, Cuerva *et al.* described several Pt(II) metallomesogens by exploring the capacity of some metal ions to induce self-assembly *via* intermolecular Pt⋯Pt interactions, generating aggregates and emission changes in solution.^126^ Only Hg^2+^ caused a notable emission change in the unsymmetrical Pt(ii) complex having pyridine 93. The emission band at 500 nm was quenched, and the minimum intensity was achieved by adding 1 equiv. of Hg^2+^ in dichloromethane (DCM). Hg^2+^ interacts with the axial position of 93 (93-Hg^2+^ 1 : 1), but an interaction with the pyrazolic N of 93 is also suggested (Fig. 32a). In contrast, Hg^2+^ ions resulted in a notable increase in the emission intensity for isoquinoline derivative 94 in a 1 : 1 ratio (Fig. 32b). In this case, the emission maximum exhibited a redshift from 527 to 644 nm (*λ*_exc_ = 365 nm), from a low greenish emission to bright red. This was attributed to the existence of metal-MLCT excited states and the intermolecular Pt⋯Pt interactions in a sandwich-type conformation.

It is important to note that other metal ions interact with probe 94, but a strong quenching effect in its natural greenish emission was observed. The result obtained in the recognition of Hg^2+^ using both probes is mainly because the coordination of Pt with the ligands prevents the formation of aggregates in the presence of the analyte. However, the additional intermolecular π⋯π interactions between the isoquinoline rings in 94 are an additional explanation for the stabilization of the Pt⋯Pt aggregates. The photophysical results revealed that these luminescent Pt(II) compounds are excellent candidates as dyes for the fabrication of several stimuli-responsive luminescent materials.^126^

## 4. Some porphyrin-based probes

The tetrameric structure of pyrrole, known as porphyrin, is a versatile structural motif; consequently, it has been extensively studies in different areas of science, even in the ion sensing field,^127–131^ and frequently in cation^129–131^ recognition. Accordingly, we mention this important macrocyclic NHC concerning its application for the detection of cyanide^127,128^ and mercury(ii)^129^ considering that they are hazardous. In general, porphyrins have high absorption in the red visible spectrum region and are potential dyes for sensitizers in photodynamic therapy; similarly, the inclusion of chromophore groups in the porphyrinic ring increases its photophysical versatility, causing significant changes in the wavelength its respective derivatives can absorb.^1,21,127–131^

**Fig. 33:**
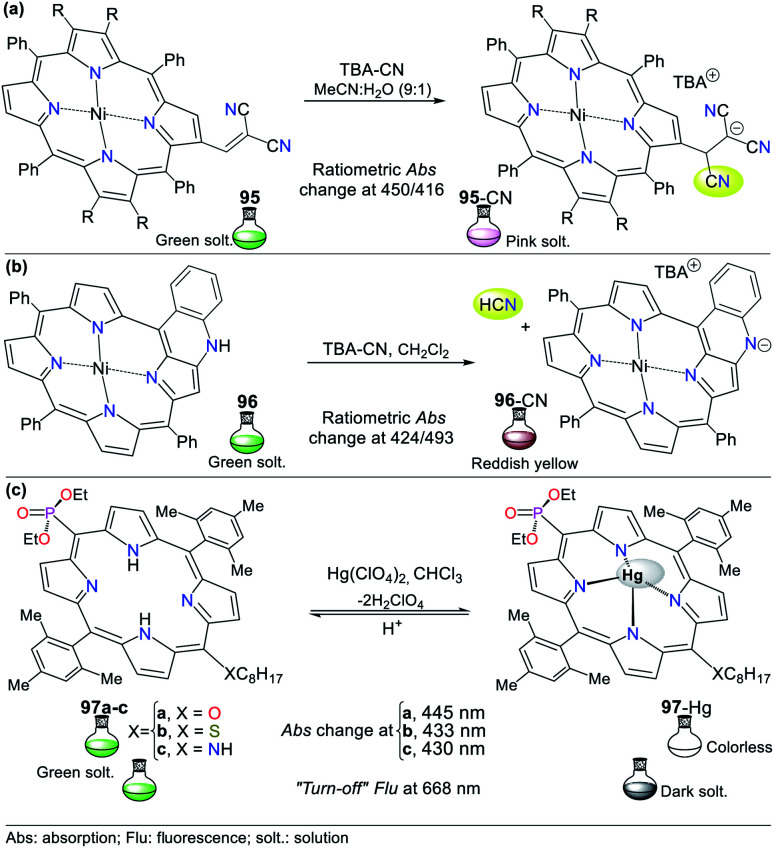
Porphyrins for the detection of CN and Hg (a) 95, (b) 96, and (b) 97.

Regarding the anion, porphyrin derivatives 95 as chemodosimeters for CN^−^ were reported by Chahal and Sankar in 2015. They obtained dicyanovinylidene-appended β-substituted porphyrins *via* Vilsmeier–Haack formylation followed by a Knoevenagel condensation reaction between the aldehyde obtained and malononitrile to obtain the dicyanovinyl derivative. The β carbon is an electrophilic center, where cyanide anions can perform a nucleophilic attack *via* design strategy **I**.^127^ This probe has essential characteristics that favor the charge transfer process, for instance, the quasi-planar conformation confirmed by crystallographic studies. Simultaneously, this structure has extended pi conjugation between the pyrrole ring and the olefinic substituent. Upon the addition of CN^−^ to a toluene solution, the color of the probe changed from green to light pink, as evidenced by the naked eye. The photophysical studies showed that the intensity bands of the bands at 385 nm, 450 nm, and 602 nm of the free sensor gradually decreased with the addition of CN^−^, while two new bands appeared at 416 nm and 534 nm. The stoichiometry of the nucleophilic addition reaction is 1 : 1. The LOD of this chemodosimeter is 0.023–0.082 ppm, which is lower than that of other reported porphyrins (Fig. 33a).

In 2020, Dar and Sankar obtained fused porphyrin 96 as a probe for the detection of cyanide.^128^ Upon adding the anion, it interacts with the acidic N–H proton, resulting in its deprotonation by strategy **III** for sensing CN^−^. This was confirmed *via*^1^H-NMR titration experiments, showing that the N–H proton at 9.22 ppm disappeared during the titration, congruent with the probe structure. It has no other reactive points towards anions. In this case, the negative charge is stabilized *via* conjugation throughout the molecule. The absorption spectrum showed a band at around 424 nm, which decreased with the addition of CN^−^ and blue-shifted to 410 nm. At this point, a new band appeared at 493 nm. The probe was tested against other anions, and it showed no interference except for fluoride. The naked eye evidenced these absorption changes due to the color change from green to reddish-yellow. Notably, this probe was shown to be reversible by washing it with distilled water. After cleaning the sensor, its color changed back to green and its absorption spectrum changed, given that water serves as a protonating agent and dissolves the anion. Finally, the calculated LOD was 2.13 ppm (Fig. 33b).

Regarding the use of porphyrin derivatives for the detection of Hg^2+^, Ermakova *et al.* synthesized three amphiphilic porphyrin derivatives 97a–c substituted at the *meso*-positions for detecting this analyte.^129^ Color changes and fluorescence quenching (*λ*_exc_ = 420 nm) were observed upon the addition of Hg^2+^ and other cations to these probes (Fig. 33c). The naked eye could observe the color change in the solutions from light green to colorless only with Hg^2+^. The redshift of the Soret band and the appearance of three or four Q-bands for the complexes can explain the metal complexation. This behavior is because the bulky metal ion is located above the macrocycle plane, and the ligand is coordinated to Hg^2+^, generating stable complexes with a ratio of 1 : 1. In contrast, other metals (Cu^2+^, Zn^2+^, and Pb^2+^) were ligated to the peripheral donor sites. The formation of a neutral complex liberates two protons, but it is not possible to form porphyrin aggregates due to the presence of bulky substituents in the porphyrin macrocycle.

Monolayers of porphyrinyl-phosphonate 97a–c were transferred to a PVC support using the Langmuir–Schaefer film method. According to the analysis of the probes, it was concluded that the films were suitable for the selective spectrophotometric recognition of Hg^2+^ in an aqueous environment. The sensor treatment by an acidic solution led to the removal of the Hg^2+^ ions, and these films could be regenerated for at least five times without loss in their efficiency. The heteroatom substituent in 97 (O, S, or NH) had a significant effect on the surface pressure-assisted supramolecular assembly of the porphyrins on solid surfaces. The LOD of these selective and reusable dual-channel probe (absorbance and fluorescence) thin-film sensors was about 10^−8^ M (2 ppb), which is lower that the recommended levels of Hg^2+^ ions in drinking water by the U.S. Environmental Protection Agency (EPA) (Fig. 33c).^129^

## 5. Conclusions and perspectives

Aza-heterocyclic compounds play a significant role in the design of probes to detect cyanide and mercury. In this case, 5- and 6-membered rings prevail in the design of probes due to their resonance effects and heteroaromaticity, allowing exceptional photophysical responses in the sensing process. Besides, the presence of N–H groups in the rings favors multiple structures with some strategic pathways such as ESIPT. Chemosensors allow the detection of ions, affording analytical methods with fewer limitations and lower costs than other approaches. With the probes considered herein, toxic and poisonous ions are quickly identified *via* colorimetric and/or fluorimetric “*turn-on*” or “*turn-off*”. Remarkably, in almost all the recently published works, tetrabutylammonium cyanide (TBACN) salt was used as the primary CN^−^ source due to its practical benefits. In addition, the characteristic complexing ability of N-heterocycles makes them highly specific for the recognition of Hg^2+^ in nature.

After analyzing the examples studied in this contribution, it was possible to qualitatively determine that Hg^2+^ tends to form stable complexes having 5- or 6-membered rings with N, O, and S atoms, mainly with a ligand–Hg 1 : 1 stoichiometry. Through this review, all the remarkable properties of a wide variety of probes could be compiled as promising tools for the detection of Hg^2+^ in the biological and environmental fields and real samples. The experimental results observed herein show a positive future for the design of N-heterocycle-based sensors with high sensitivity and selectivity towards Hg^2+^. Further, novel ways are revealed to more easily develop simple, selective, highly sensitive, low-cost, and reversible probes bearing different fused and non-fused NHCs conjugated with other fluorophores to have a greater diversity of coordination sites, and thus detect toxic ions such as Hg^2+^.

Ultimately, we showed that N-heterocycles are valuable molecules in the design of chemosensors due to their advantages such as synthetic versatility, use in wide pH range, the low detection limit, and the characteristic characterization of their sensing mechanism is straightforward. Nevertheless, the design of new probes with lower LOD, more selectivity towards some interferents (*e.g.*, F^−^, SH^−^, and some metal ions), the possibility of using 100% aqueous medium in the detection, and reversibility are still crucial to give a closer approach to real samples. Additionally, although many published works on fluorescent and colorimetric chemosensors are small-molecule-based, several of them have been developed without a rational design; consequently, a good look at this review can significantly improve this issue.

## Authors contributions

The four individuals listed as authors have contributed substantially to the development of this review, and no other person was involved with its development. The contribution of authors is as follows:

Miss María C. Ríos. Papers analysis involving azole derivatives in cyanide detection and original draft composition.

Mr Néstor F. Bravo. Literature analysis involving all described probes in mercury(ii) recognition and original draft composition.

Mr Christian C. Sánchez. Papers analysis on pyridine derivatives and metal complexes in cyanide sensing and original draft composition.

Prof Jaime Portilla. Literature investigation, supervision, writing, manuscript review and editing, conceptualization, and resources.

## Conflicts of interest

The authors declare no competing financial interest.

## Supplementary Material
